# Managing missing items in the Fagerström Test for Nicotine Dependence: a simulation study

**DOI:** 10.1186/s12874-022-01637-2

**Published:** 2022-05-20

**Authors:** Shannon L. Gutenkunst, Melanie L. Bell

**Affiliations:** 1 University of Arizona, Statistics Graduate Interdisciplinary Program, Tucson, AZ USA; 2 University of Arizona, Statistics Consulting Lab, Health Sciences & Bio5 Institute, Tucson, AZ USA; 3 Department of Epidemiology and Biostatistics, Mel and Enid Zuckerman College of Public Heath, University of Arizona, Tucson, AZ USA

**Keywords:** Missing data, Item level missingness, Item nonresponse, Imputation, Questionnaire, Scoring

## Abstract

**Background:**

The Fagerström Test for Nicotine Dependence (FTND) is frequently used to assess the level of smokers’ nicotine dependence; however, it is unclear how to manage missing items. The aim of this study was to investigate different methods for managing missing items in the FTND.

**Methods:**

We performed a simulation study using data from the Arizona Smokers’ Helpline. We randomly sampled with replacement from the complete data to simulate 1000 datasets for each parameter combination of sample size, proportion of missing data, and type of missing data (missing at random and missing not at random). Then for six methods for managing missing items on the FTND (two involving no imputation and four involving single imputation), we assessed the accuracy (via bias) and precision (via bias of standard error) of the total FTND score itself and of the regression coefficient for the total FTND score regressed on a covariate.

**Results:**

When using the total FTND score as a descriptive statistic or in analysis for both types of missing data and for all levels of missing data, proration performed the best in terms of accuracy and precision. Proration’s accuracy decreased with the amount of missing data; for example, at 9% missing data proration’s maximum bias for the mean FTND was only − 0.3%, but at 35% missing data its maximum bias for the mean FTND increased to − 6%.

**Conclusions:**

For managing missing items on the FTND, we recommend proration, because it was found to be accurate and precise, and it is easy to implement. However, because proration becomes less accurate with more missing data, if more than ~ 10% of data are missing, we recommend performing a sensitivity analysis with a different method of managing missing data.

**Supplementary Information:**

The online version contains supplementary material available at 10.1186/s12874-022-01637-2.

## Background

The Fagerström Test for Nicotine Dependence (FTND) is a commonly used questionnaire that measures physiological dependence on nicotine [1], and a review of 26 papers on the psychometric properties of the FTND found that it was reliable in different populations [2]. The FTND is used by researchers in observational studies and clinical trials, and it has positive associations with important smoking variables, such as salivary cotinine concentration used for biochemical verification of cessation [3]. The FTND is also used by clinicians, to decide nicotine replacement therapy dosages. Table 1 presents the six FTND questions and their response options. The total FTND score is calculated by summing the scores from the individual items. Total possible FTND scores range from 0 to 10, with higher scores indicating higher levels of nicotine dependence. Other popular questionnaires have published recommendations for how to manage missing items [4–6]; however, there are no published recommendations for how to manage missing items in the FTND.

**Table 1:** Descriptive statistics for the Fagerström Test for Nicotine Dependence (FTND)

FTND item number	Question with answer scoring	Missing N (%)	Mean (SD)	Spearman rank correlation of item with total of remaining items
1	How soon after you wake up do you smoke or use tobacco? 0 = after 60 min. 1 = 31-60 min. 2 = 6-30 min. 3 = within 5 min.	269 (0.7%)	2.0 (1.0)	0.48
2	Do you find it difficult to refrain from smoking in forbidden places (e.g., church, library, cinema)? 0 = No 1 = Yes	378 (1.0%)	0.3 (0.4)	0.26
3	Which cigarette would you hate to give up? 0 = any other time 1 = first in the morning	386 (1.0%)	0.6 (0.5)	0.35
4	How many cigarettes per day do you smoke? 0 = 10 or less 1 = 11-20 2 = 21-30 3 = 31+	159 (0.4%)	0.9 (0.8)	0.36
5	Do you smoke more frequently during the first hours after waking than during the rest of the day? 0 = No 1 = Yes	402 (1.0%)	0.4 (0.5)	0.31
6	Do you smoke if you are so ill that you are in bed most of the day? 0 = No 1 = Yes	388 (1.0%)	0.5 (0.5)	0.31
Total FTND score	Possible range: 0-10. Higher scores indicate higher nicotine dependence: 0-2 Very low 3-4 Low 5 Medium 6-7 High 8-10 Very high	408 (1.1%)	4.7 (2.3)	-

Descriptive statistics for the Fagerström Test for Nicotine Dependence (FTND) for the 38,742 clients enrolled in Arizona Smoker’s Helpline (ASHLine) from January 3, 2011 to June 23, 2016 and who answered at least one FTND question. *FTND* Fagerström Test for Nicotine Dependence, *ASHLine* Arizona Smoker’s Helpline, *min.* minutes, *SD* standard deviation

The main statistical problems caused by missing items on the FTND (or any questionnaire) are bias and loss of power. Bias can occur if the outcomes of smokers who skipped questions are different than those who completed them all. For example, if heavy smokers are embarrassed about their reliance on cigarettes, they may be more likely to skip questions on the FTND; if data from those smokers are ignored in the analysis, then the estimated mean FTND score for a sample of smokers would be less than the true FTND score if the heavy smokers had answered all items. Loss of power is caused by reduced sample size when only data from smokers who answered all questions are analyzed.

Most researchers with missing items on the FTND have either not reported how they managed missing items or performed complete case analysis (CCA; where only data from respondents who have no missing items is used in the analysis, which has most frequently been used on questionnaires in epidemiology research [7]). A preliminary review of the literature identified many studies where the method of managing missing items was not stated, one study that explicitly applied CCA to missing items on the FTND [3], and two other studies that applied the following rule for handling missing items on the FTND that we will call the drop one method: if only one item was missing, the FTND total score was calculated without it (i.e., the missing item was assumed to have a score of zero); if more than one item was missing, the FTND total score was coded as missing [8, 9]. Later, a more systematic search of the literature was performed: PubMed was searched for “Fagerström Test for Nicotine Dependence” on March 7, 2022, and the 50 most recent manuscripts were checked to determine the method used for handling any missing items on the FTND (Additional file 1). We found that 18/50 (36%) manuscripts had an unknown method for managing missing items, 11/50 (22%) applied CCA, and 1/50 (2%) applied a modified drop one rule [like drop one, but they also did not calculate a total score if item 4 (number of cigarettes smoked per day) was missing] [10]. An additional 16/50 (32%) had complete data (with no missing FTND items), and for 4/50 (8%) a method of managing missing items was not applicable because, for example it was a review. Other methods commonly used for managing missing items on questionnaires include the following single-imputation methods: item mean imputation (imputing the value of a missing item with the mean of the observed values for that item from other participants in the sample), proration (imputing the value of a participant’s missing item based on their answers to other items in the questionnaire), and nearest neighbor hot deck imputation (imputing the values of missing items for each participant with values from a participant that otherwise has similar characteristics to the participant with missing items). Methods that use no imputation can lead to bias and loss of power. Single imputation methods can underestimate the variability of estimates which can affect statistical tests, and they can potentially lead to bias and loss of power as well.

Another method for imputing missing data is multiple imputation (MI), which is often considered to be the “gold standard” for imputation. In MI, an algorithm is applied to impute values for missing items, by drawing from a distribution of plausible values determined from other variables, multiple (often ~ 20) times, and the results are combined according to Rubin’s rules [11], which has the advantage of incorporating the uncertainty due to imputation. However, MI has disadvantages, including that it is harder to implement than single-imputation methods. If a parametric MI approach is used, there is potential for non-convergence if more than one variable has missing data, and it also requires the assumption that an item’s missingness does not depend on what would have been the true value. For missing items in questionnaires, MI is also difficult to implement in practice, because it is not clear when MI should be applied if other variables also have missing values, and the researcher wants to use the total questionnaire score in analysis. Because of these disadvantages of MI especially for missing items in questionnaires, and because using no imputation (e.g., CCA or drop one) for missing questionnaire items is still unfortunately quite common, this study focused on comparing easily implemented single-imputation methods against each other and against methods that do not use imputation for missing items on the FTND.

The objective of this paper was to investigate easily implemented approaches to manage item nonresponse on the FTND by running simulations on data from smokers who completed the FTND. Specifically, we investigated six approaches for managing missing items for two samples sizes, several different proportions of missing data at both the subject and item levels, and two missing data mechanisms. To evaluate these methods, we assessed the accuracy and precision of the descriptive variable total FTND score and the association variable of a regression coefficient for total FTND score regressed on a covariate.

## Methods

**Fig. 1:**
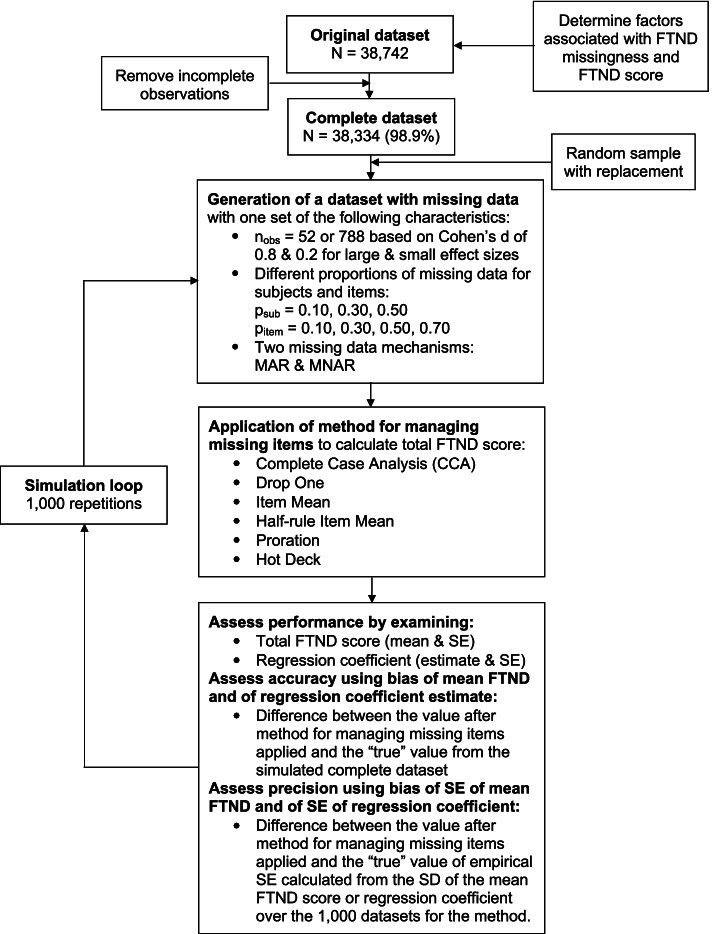
Simulation study design

We performed a simulation study using data from callers to the Arizona Smoker’s Helpline who answered at least one question on the FTND. First, we removed incomplete observations from that real data, so that we could control the missing data mechanism and calculate the bias because the “true” value was known. Then, we drew random samples with replacement to generate 1000 datasets for each parameter combination: two sample sizes, several proportions of missing data for subjects and for items, and two missingness mechanisms. We then applied six different methods for managing missing items to calculate the total FTND score, and we evaluated the performance of the methods for both the total FTND score and the coefficient for the total FTND score regressed on a covariate. Figure 1 gives an overview of these steps in the simulation study, and they are detailed below. The simulation was coded in R version 3.6.2 [12] and used the tidyverse R package [13]; the simulation code is available in Additional files 2, 3, 4 and 5.

### Data source

We started with data from 49,284 clients enrolled in ASHLine (Arizona Smoker’s Helpline; the state of Arizona’s quitline) from January 3, 2011 to June 23, 2016 who received standardized tobacco cessation protocols. Data for this study were collected over two time periods: the first phone call at time of enrollment (including demographics), and the second phone call (first coaching call, including answers to FTND items). The data used in this study starts with the subset of 38,742 clients who answered at least one of the FTND questions during the second phone call. Clients were 56.7% female (42.4% male; 0.9% missing) and ranged in age from 15 to 93 years with a mean age of 49.2 years [standard deviation (SD): 14.1 years; 0.9% missing]. Clients were asked about their race/ethnicity on two separate questions, and clients were 72.2% White, 16.9% Hispanic (65.7% non-Hispanic; 17.4% missing), 7.1% Black or African American, 6.6% other race (14.1% missing). For education, 15.4% of clients had less than high school, 29.6% had high school, 34.0% had some above high school, and 18.0% had a college degree (3.0% missing). Out of the 38,742 clients, 38,334 (98.9%) answered all the FTND items.

### Simulation sample sizes

We started with the subset of 38,334 clients who completed all items on the FTND. Then we sampled with replacement to select a random sample for a simulation. We chose a small sample size of n_obs_ = 52 and a large sample size of n_obs_ = 788 based on detecting, respectively, a large effect size corresponding to Cohen’s standardized effect [14] d = 0.8 and a small effect size corresponding to Cohen’s d = 0.2, with 80% power for a two-sided t-test with Type I error of 0.05.

### Missingness mechanisms

To imitate missingness patterns seen in questionnaire responses where missing items appear in only a subset of subjects, only certain subjects were eligible for item-level missingness with probability p_sub_. Which subjects were eligible for item-level missingness depended on the missing data mechanism, as described below. Then item-level missingness was assigned to eligible subjects with probability p_item_ using the Bernoulli distribution: for each subject eligible for missingness, a 0 or 1 was generated for each item, with 1 being generated with probability p_item_. Items assigned the value 1 were changed to missing. To span a range of missingness rates from the small level seen in our ASHLine data to larger rates to distinguish the performance of different methods of managing missing items, we chose to simulate with values of p_sub_ = 0.1, 0.3, 0.5 and p_item_ = 0.1, 0.3, 0.5, 0.7, resulting in overall proportions of missing items ranging from 0.01 to 0.35.

Investigating different causes of missingness (i.e., missingness mechanisms) is important, because the performance of different methods for managing missing items depends on the underlying reasons for the data being missing. Little and Rubin [15] defined three categories of missingness mechanisms. Data are missing completely at random (MCAR) if the probability of an item being missing (i.e., it’s missingness) is independent of the subject’s missing or observed characteristics. Data are missing at random (MAR) if an item’s missingness depends on the subject’s observed characteristics (note: some consider this covariate-dependent missingness). Data are missing not at random (MNAR) if an item’s missingness depends on what would have been the true value. In this study, we implemented MAR and MNAR missingness mechanisms, omitting MCAR missingness because its main effect is just to reduce the sample size, and MAR and MNAR missingness are likely more realistic.

The MAR missingness mechanism was carried out by choosing subjects to be eligible for item-level missingness based on two variables that were associated at *p* < 0.05 in our original sample (*N* = 38,742) with FTND missingness using single-variable logistic regression and with the FTND value itself using single-variable regression. The two variables were gender (0 = female; 1 = male) and the answer to the question, “If you smoke at home, where?” with possible answers 0 = No; 1 = Yes (outside), and 3 = Yes (inside). Subjects were eligible for missingness with a probability (Pr) determined by the following logistic regression model, where *smoke* _ *where*_1_ and *smoke* _ *where*_2_ are indicator variables for smoking outside at home and smoking inside at home, respectively:

$$logit\Pr (missing)={\beta}_0+{\beta}_1\ast gender+{\beta}_2\ast smoke\_ wher{e}_1+{\beta}_3\ast smoke\_ wher{e}_2$$

Additionally, we fixed the coefficients *β*_1_, *β*_2_, and *β*_3_ from logistic regression of FTND missingness on gender and the two smoke_where variables in the original dataset (*N* = 38,742) as follows: *β*_1_ = 0.20 (corresponding to an odds ratio OR = 1.22), *β*_2_ =  − 2.12 (corresponding to OR = 0.12), and *β*_3_ =  − 1.99 (corresponding to OR = 0.14). Thus, males were more likely to be eligible for missingness than females, and those who answered that they did not smoke at home were more likely to be eligible for missingness than those who answered that they did smoke at home (either inside or outside). We then chose the value of *β*_0_ empirically to make approximately p_sub_ subjects eligible for missingness. Finally, item-level missingness was performed within those subjects with probability p_item_ as detailed above.

The MNAR missingness mechanism was implemented by choosing subjects to be eligible for item-level missingness based on their total FTND score, with a probability determined by the following logistic regression model:

$$logit\Pr (missing)={\beta}_0+{\beta}_1\ast FTND$$

where we fixed the coefficient *β*_1_ = 0.2 (corresponding to OR = 1.22 for a 1-point increase in FTND), so that subjects exhibiting higher nicotine dependence were more likely to be eligible for missingness. The value of *β*_0_ was again chosen empirically to qualify roughly p_sub_ subjects for missingness. Finally, item-level missingness was carried out within those subjects with probability p_item_ as described above.

### Methods for managing missing items

**Complete case analysis (CCA)**, also referred to as listwise deletion, was performed by only calculating the total FTND score for subjects who had no missing items.

The **drop one** method was implemented as follows: if a subject had only one item missing, their total FTND score was calculated without it. Thus, the missing item was assumed to have a value of zero. If a subject had more than one item missing, their total FTND score was coded as missing.

**Item mean** imputation was carried out by imputing a missing item with the mean score for that item for all participants who answered it. **Half-rule (HR) item mean** imputation was performed like item mean imputation, but only if at least half (3) of the items on the FTND were non-missing for a subject (otherwise the total FTND score was coded as missing).

**Proration**, sometimes called person or subject mean imputation, involves imputing the value of a subject’s missing item based on their answers to other items in the questionnaire. Imputation with proration was only performed following the half rule suggested for other questionnaires [4, 6]: if at least half (3) of the items on the FTND were non-missing for a subject, then the item was imputed (otherwise the total FTND score was coded as missing). For the FTND, items 1 and 4 have possible points (0, 1, 2, 3); the other four items all have possible points (0, 1). Thus, to weight items appropriately for proration, imputed item values were calculated as follows before summing values from all items to obtain the total FTND score:

$$\mathrm{Items}\ 1\;\&\;4:\kern0.5em imputed\ item\ value=3\ast \left(\frac{total\ score\ of\ questions\ subject\ answered}{total\ possible\ score\ of\ questions\ subject\ answered}\right)$$

$$\mathrm{Items}\;\ 2,\;3,\;5,\;6: imputed\ item\ value=\frac{total\ score\ of\ questions\ subject\ answered}{total\ possible\ score\ of\ questions\ subject\ answered}$$

K-nearest neighbor **hot deck** imputation consisted of imputing the values of missing items for each subject with values from a subject with data for those items that in other respects was like the subject with missing items. First, for each subject with one or more missing FTND items (called a recipient), the predictors of gender, the smoking allowed in home variable “Is smoking allowed in your home?” with responses 0 = No and 1 = Yes, and the subject’s non-missing FTND items were used to calculate Gower’s distance [16] between subjects to determine the k most similar subjects with non-missing data for those items. Second, a single donor was randomly chosen from those k subjects to donate imputed values to the recipient. Hot deck was implemented using the *simputation* R package [17] with k = 5 and pool = “multivariate”, so a pool of five donors was generated for each recipient, and if a recipient had more than one missing item, all imputations were provided by a single donor.

### Performance measures

We assessed accuracy by estimating the bias of the descriptive parameter of the mean total FTND score itself and of the regression coefficient (association parameter) for the total FTND score in single linear regression on the explanatory variable, “Is smoking allowed in your home?” with responses 0 = No and 1 = Yes. We estimated the bias of each quantity for each simulated dataset by calculating the difference between its value after a method for managing missing items was applied ($\hat{\theta}$) and its “true” value from the same simulated dataset before missingness was generated (*θ*): $\hat{\theta}-\theta$; the percent bias was calculated by dividing the bias by the “true” value and multiplying by 100: $\frac{\hat{\theta}-\theta }{\theta}\ast 100.$ Then, we calculated the mean and 95% Monte Carlo confidence interval (CI) over the 1000 datasets.

We assessed precision for the FTND by estimating the bias of the standard error (SE) of the mean FTND score compared to the “true” empirical SE calculated from the standard deviation (SD) of the mean FTND score over the 1000 datasets for each missingness mechanism and method. Similarly, we assessed precision for the regression coefficient by estimating the bias of its SE compared to its “true” empirical SE calculated from the SD of the regression coefficient over the 1000 datasets for each missingness mechanism and method [18]. Zero bias for these quantities indicates high precision. We calculated percent bias as above, as well as the mean and 95% CI over the 1000 datasets.

A clinically important level of bias for the mean FTND score was considered to be 1 for two reasons: (1) a difference of 1 point can make the difference between distinct categories of nicotine dependence [e.g., 1 point can mean the difference between a subject being categorized as having medium (score 5) versus high (scores 6 or 7) nicotine dependence], and (2) a minimal important difference of 10% (equal to 1 point on the FTND) may be used in patient-reported outcome questionnaires [19].

## Results

### Descriptive statistics of original sample

Table 1 gives descriptive statistics of the original sample. The mean FTND score was 4.7 with standard deviation 2.3. The nicotine dependence of ASHLine clients as measured by the FTND varied across the entire spectrum (18.0% very low dependence, 24.4% low, 16.8% medium, 28.7% high, and 11.0% very high; see Table 1 for definitions of dependence ranges), with 1.1% of clients not reporting at least one FTND item. The low missingness rate for the FTND may be because ASHLine collects this data over the phone, which allows phone coaches to immediately follow up with a smoker if they originally do not answer an FTND question. Missing FTND item rates ranged from 0.4 to 1.0%. Spearman rank correlations of each item with the total of remaining items were moderate (0.26-0.48); correlations between pairs of FTND items ranged from small (0.07) to moderate (0.39). Cronbach’s alpha for the FTND in the ASHLine data was 0.59, indicating moderate internal consistency, and within the range of 0.55 to 0.74 found in a summary of 14 other studies using the FTND [2].

### Simulation results

**Fig. 2:**
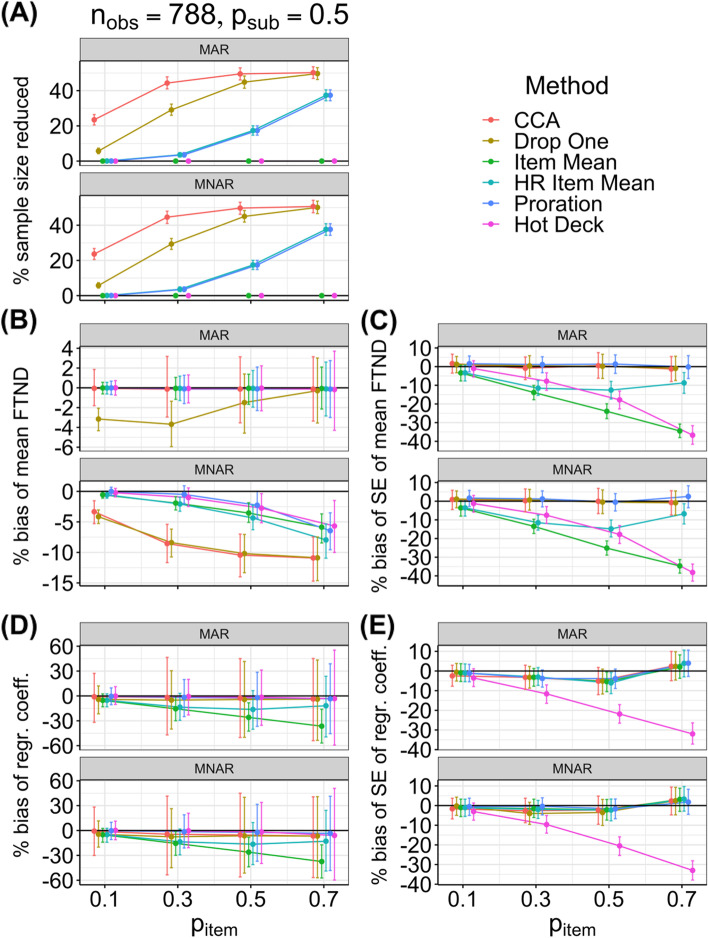
Plots of simulation results. For the large sample size (n_obs_ = 788) with probability of subject-level missingness p_sub_ = 0.50, plots of estimates of the (**A**) percent sample size was reduced and (**B-E**) performance measures (mean with 95% Monte Carlo confidence interval) for each method against the probability of item-level missingness (p_item_) for both missingness mechanisms (MAR and MNAR). CCA = Complete Case Analysis; FTND = Fagerström Test for Nicotine Dependence; HR = Half-Rule; MAR = missing at random; MNAR = missing not at random; regr. Coeff. = regression coefficient; SE = standard error

Figure 2 presents plots for both missingness mechanisms (MAR and MNAR) of estimates of the (A) percent the sample size was reduced and (B-E) performance measures for each method against item-level missingness p_item_ for the large sample size (n_obs_ = 788) and subject-level missingness p_sub_ = 0.50. Additional file 6 presents full results with such plots for each sample size and value of subject-level missingness. Going from the small (n_obs_ = 52) to the large (n_obs_ = 788) sample size resulted in similar point estimates for our performance measures but smaller Monte Carlo 95% CIs; for example, the most extreme value of bias for the mean FTND was for CCA with the most amount of MNAR missingness, with the same point estimate for both sample sizes for percent bias of − 11% (corresponding to absolute bias of − 0.5), but with a 95% CI at n_obs_ = 52 of (− 26%, 2%), corresponding to (− 1.2, 0.1) and a 95% CI at n_obs_ = 788 of (− 15%, − 7%), corresponding to (− 0.7, − 0.4). Thus, even the most extreme value of bias for any method for the mean FTND did not result in a clinically meaningful amount of bias of one point (the 95% CI for n_obs_ = 52 barely crossed that threshold). However, more bias occurred for other measures, as discussed below. All Monte Carlo 95% CIs presented below are for n_obs_ = 788.

The sample size plot (Fig. 2A) shows that the sample size was not affected for methods that imputed all item values (hot deck and item mean). The sample size decreased with increasing missingness for the other methods, with CCA having the largest decrease, followed by drop one, and finally by proration and half-rule item mean (tied, because they used the same half rule for deciding whether to impute an item).

The plots of percent bias of the mean FTND (Fig. 2B) show that the results depended on the type of missingness. For MAR data (Fig. 2B, top panel), all methods except drop one appeared basically unbiased (bias ≤ 0.2% in magnitude) at all levels of subject and item missingness. However, drop one had a negative bias (as expected) for low levels of item missingness [maximum bias: − 4% with 95% CI (− 6%, − 1%), corresponding to − 0.17 (− 0.29, − 0.06)]. For MNAR data (Fig. 2B, bottom panel), all methods produced negative biases, and the magnitude of the bias increased with the amount of missing data. CCA and drop one behaved similarly and had the most bias; however, even for these cases the levels of bias were not high (maximum bias of − 11% as discussed above). Proration and hot deck behaved similarly and generally had the least bias: maximum bias for proration − 6% (− 10%, − 4%) corresponding to − 0.3 (− 0.5, − 0.2); maximum bias for hot deck − 6% (− 10%, − 2%) corresponding to − 0.3 (− 0.5, − 0.1). Because proration performed best when considering all measures, we note that proration’s bias at 9% missing data (p_sub_ = 0.3, p_item_ = 0.3) was only − 0.3% (− 1.4%, 0.8%) corresponding to − 0.02 (− 0.07, 0.04).

For percent bias of the SE of the mean FTND (a measure of precision), results for each method were similar for both types of missing data (Fig. 2C). CCA, drop one, and proration had bias of the SE of the mean FTND ≤ 6% in magnitude for all amounts of missing data and thus were relatively precise. Item mean and hot deck had the most bias (and thus were least precise), and the bias was negative, with the magnitude increasing with the amount of missing data to a maximum bias of − 35% (− 39%, − 31%) corresponding to − 0.03 (− 0.04, − 0.03) for item mean and − 38% (− 43%, − 34%) corresponding to − 0.05 (− 0.06, − 0.05) for hot deck.

The plots of percent bias of the regression coefficient (Fig. 2D) show that the results did not meaningfully depend on the type of missingness. Biases were generally negative, and item mean had the most bias, with the magnitude of the bias increasing with the amount of missingness: maximum bias − 37% (− 57%, − 17%) corresponding to − 0.27 (− 0.45, − 0.10). Proration and hot deck had the least bias, with more missing data generally corresponding to more bias: proration’s maximum bias was − 4% (− 36%, 27%) corresponding to − 0.02 (− 0.29, 0.26), and hot deck’s maximum bias was − 6% (− 60%, 51%) corresponding to − 0.04 (− 0.42, 0.31). Because proration performed best when considering all measures, we note that proration’s bias at 9% missing data (p_sub_ = 0.3, p_item_ = 0.3) was only − 1.2% (− 17.9%, 14.4%) corresponding to 0.05 (− 0.12, 0.09).

For the measure of precision of the percent bias of the SE of the regression coefficient (Fig. 2E), results for each method were similar for both types of missing data. All methods except hot deck had bias ≤ 6% in magnitude for all amounts of missing data and thus were relatively precise. Hot deck had a negative bias that increased in magnitude with the amount of missing data up to a maximum amount of bias of − 33% (− 38%, − 28%) corresponding to − 0.08 (− 0.09, − 0.07); thus, hot deck was imprecise.

## Discussion

In this simulation study we compared six methods for managing missing items in the FTND. Of all the methods we compared, proration performed the best overall. Proration was typically the most accurate, having the least biased mean FTND and regression coefficient. Proration became less accurate for increasing amounts of missing data: at 9% missing data proration’s maximum bias for the mean FTND was only − 0.3% and for the regression coefficient − 1.2%; however, at 35% missing data its maximum bias for the mean FTND increased to − 6% and for the regression coefficient to − 4%. Proration was also precise, having a maximum bias of the SE of the mean FTND and of the SE of the regression coefficient of 6%, although there was not a clear trend with the amount of missing data. Thus, of the six methods we compared for managing missing items in the FTND, we recommend proration for its accuracy and precision, and because it is easy to implement.

The conclusions of our study generally agree well with those of other studies that evaluated methods for managing missing items in other questionnaires. For example, for missing items on the Functional Assessment of Cancer Therapy General (FACT-G), Fairclough and Cella did a simulation study with 1.7 and 8% missing data, and they recommended imputing with the mean of the completed items in a subscale when > 50% of items are completed (similar to our proration), because it was the most unbiased and precise method [4]. Additionally, for missing items on the Medical Outcome Study 36-item Short-Form health survey (SF-36), Peyre et al. ran a simulation study with 3, 6, and 9% missing data, and they found that MI and full information maximum likelihood (FIML) had better accuracy and precision than person mean score (similar to our proration); however, the person mean score was associated with insignificant bias (< 2%) in all cases, and they recommended person mean score for its ease of use when missingness < 10% [5]. Further, for missing items on the Hospital Anxiety and Depression Scale (HADS), Bell et al. performed a simulation study with a range of missing data from 2 to 25%, and they recommended either the subject or subscale mean (similar to our proration) or MI, preferring subject or subscale mean for their ease of use [6].

The relatively low inter-item correlations for the FTND (range: 0.07-0.39) might be expected to negatively affect proration. However, proration has been found to be an effective imputation strategy in another questionnaire with relatively low inter-item correlations. The Myeloproliferative Neoplasms 10-item total symptom score (MPN-10) also had relatively low inter-item correlations (range: 0.19-0.58), and Mazza et al. compared proration and MI in their dataset where 12% of respondents had skipped at least one item, and they found that proration and MI produced similar results [20].

### Strengths and limitations

A strength of this study is that the original ASHLine dataset was large (*N* = 38,742) with data from a diverse set of individuals with a wide range of ages, several race/ethnicities, and a wide range of educational levels, suggesting the results may be generalizable. An additional strength of basing simulations on the ASHLine dataset is that the simulated situations mirror actual research circumstances; thus, they give a reasonable idea of the size of the effects on the results. Further strengths of this simulation study were that it considered two different missingness mechanisms for two sample sizes, it included a wide range of missing data (from 1% up to 35%), and it investigated accuracy and precision not only for using the FTND as a descriptive statistic but also for using it in an analysis.

A limitation of this study is that it assumed that each FTND item was equally likely to be missing. This assumption seemed reasonable because item missingness rates all ranged between 0.4 and 1.0%. However, in our original dataset (*N* = 38,742) items 1 and 4, which can contribute up to three points each to the total FTND score, had missingness rates of 0.7 and 0.4% respectively, whereas all the other items, which can contribute only up to one point each to the total FTND score, had missingness rates of 1.0%. Thus, this simulation study may be overestimating the effect of missingness.

Another limitation is that our original dataset only had 1.1% missing FTND scores. Thus, the relationships between variables and missingness in the original dataset that we used to create MAR simulated data may not apply to datasets with higher amounts of missing FTND scores.

A further limitation of this simulation study is that we only used a single dataset, data from the ASHLine. However, this study’s conclusions may be generalizable for three reasons: (1) the standard deviation of the total FTND score in this dataset of 2.3 agreed well with that from other studies (e.g., 2.32 [21], 2.43 [3], and 2.24-2.52 for different ethnicities [8]); (2) Cronbach’s alpha for the FTND in this dataset of 0.59 was within the range of 0.55 to 0.74 found in 14 others [2]; and (3) the original ASHLine dataset was large with a diverse group of respondents.

## Conclusions

In our brief review of papers that reported FTND scores, most authors did not report if they had missing items or how they managed them. Reporting the missingness rates and methods for managing missing items helps with interpreting results; authors should report them and discuss their impact on the validity of their results, justifying their choice of method for managing missing data. For using the FTND score either as a descriptive statistic or in inferential analysis, we recommend using proration, because of its accuracy, precision, and ease of use. However, because proration becomes less accurate for increasing amounts of missing data, we recommend that if the amount of missing data exceeds ~ 10%, authors should include a sensitivity analysis that uses a different imputation method. And of course, the best strategy is to prevent missing data in the first place.

## Supplementary Information

**Additional file 1.** A literature review to answer the question: How are missing items currently handled on the FTND?

**Additional file 2.** R code to clean the ASHLine data and export .rds files for use in Additional file 3 R code.

**Additional file 3.** R code to simulate data sets and apply different methods.

**Additional file 4.** R code with functions to use in the simulation, called from Additional file 3.

**Additional file 5.** Rmarkdown code for plots of FTND simulation results plotted in Additional file 6.

**Additional file 6.** Plots of FTND simulation results.

## Data Availability

Data supporting findings of this study were drawn from the Arizona Smokers’ Helpline database. Restrictions apply regarding data availability, and the data may not be publicly available. However, data may be available from the corresponding author upon reasonable request and with permission of the Arizona Smokers’ Helpline. The simulation computer code is available in Additional files 2, 3, 4 and 5.

## Abbreviations

ASHLine: Arizona Smoker’s Helpline
CCA: complete case analysis
CI: confidence interval
FACT-G: Functional Assessment of Cancer Therapy General
FIML: full information maximum likelihood
FTND: Fagerström Test for Nicotine Dependence
HADS: Hospital Anxiety and Depression Scale
HR: half-rule
MAR: missing at random
MCAR: missing completely at random
MI: multiple imputation
min.: minutes
MNAR: missing not at random
MPN-10: Myeloproliferative Neoplasms 10-item total symptom score
n_obs_: sample size
p_item_: probability of item-level missingness
Pr: probability
p_sub_: probability of subject-level missingness
regr. Coeff.: regression coefficient
SD: standard deviation
SE: standard error
SF-36: Medical Outcome Study 36-item Short-Form health survey
