# Supplementary material for: Managing missing items in the Fagerström Test for Nicotine Dependence: a simulation study
Source: BMC Med Res Methodol. 2022 May 20;22:145. doi: 10.1186/s12874-022-01637-2 (PMC9121580; doi:10.1186/s12874-022-01637-2)
Supplement: Supplementary file 1 — Additional file 1. A literature review to answer the question: How are missing items currently handled on the FTND? [file 12874_2022_1637_MOESM1_ESM.docx]

**Additional File 1: A literature review to answer the question:**

**How are missing items currently handled on the FTND?**

**“Managing missing items in the Fagerström Test for Nicotine Dependence: a simulation study”**

Shannon L Gutenkunst & Melanie L Bell

A search on PubMed for "Fagerström Test for Nicotine Dependence" on March 7, 2022 yielded 799 results. The results were sorted by most recent. Then the 50 most recent manuscripts were inspected to determine the method used for any missing items on the FTND.

**Missing data methods observed:**

**Complete case analysis (CCA):** Any cases with missing FTND items were deleted before analysis. Since this is still the most common way to deal with missing data, if the study had fewer FTND scores than participants who took the FTND, CCA was assumed unless another method was stated.

**Drop one**: Drop one method. If only one item was missing, the FTND total score was calculated without it (i.e., the missing item was assumed to have a score of zero); if more than one item was missing, the FTND total score was coded as missing.

**Drop one and exclude if missing item 4**: Like Drop one, but also do not calculate a total score if item 4 (number of cigarettes smoked per day) was missing.

**Unknown**: Unknown if they had complete data to begin with or how they handled missing FTND items.

**Not applicable (N/A)**: Not applicable, because the article was a review (3 articles), or it was written in Chinese (1 article).

**Complete data (none missing)**: The study’s FTND data were complete to begin with. It did not have any missing FTND data. If a study had FTND scores for every participant who took the FTND, complete FTND data was assumed unless another method was stated.

**Summary of results:**

| **Missing data method** | **n (%)**  **(N = 50)** |
| --- | --- |
| Complete case analysis (CCA) | 11 (22%) |
| Drop one | 0 (0%) |
| Drop one and exclude if missing item 4 | 1 (2%) |
| Unknown | 18 (36%) |
| Not applicable (N/A) | 4 (8%) |
| Complete data (none missing) | 16 (32%) |

**Note:**

A prior (less systematic) review of the literature identified one study that applied complete case analysis to missing items on the FTND (Fu et al., 2012), two other studies that applied the Drop one method (van Amsterdam et al. 2019; van Binnendijk et al. 2020), and many other studies that either had complete data or the method was unknown.

**Fu M**, Martínez-Sánchez JM, Agudo A, Pascual JA, Ariza C, Moncada A, et al. Nicotine depedence and salivary cotinine concentration in daily smokers. Eur J Cancer Prev. 2012;21:96–102.

**van Amsterdam J**, Vorspan F, Snijder MB, van den Brink W, Schene AH, Stronks K, et al. Use of the Fagerström test to assess differences in the degree of nicotine dependence in smokers from five ethnic groups: The HELIUS study. Drug Alcohol Depend. 2019;194:197–204.

**van Binnendijk S**, van Amsterdam JGC, Snijder MB, Schene AH, Derks EM, van den Brink W. Contribution of alcohol and nicotine dependence to the prevalence of depressed mood in different ethnic groups in the Netherlands: The HELIUS study. J Dual Diagn. 2020;16:271–84.

**Detailed summary:**

| **Number** | **Title and Author(s)** | **Journal Reference** | **Missing data method** | **Notes** |
| --- | --- | --- | --- | --- |
| 1 | Examining the role of mitochondrial genetic variation in nicotine dependence. Giannoulis SV, Chenoweth MJ, Saquilayan P, Tyndale RF, Lerman C, Kennedy JL, Zawertailo L, Gonçalves V. | Psychiatry Res. 2022 Feb 17;310:114452. doi: 10.1016/j.psychres.2022.114452 | CCA | CCA assumed. If any of the FTND data was missing, it was handled with complete case analysis, but methods not explicitly stated for FTND:  “An additional 6 individuals were removed due to missing phenotypic data.” FTND was one of several phenotype variables. |
| 2 | Smoking and nicotine dependence in relation to depression, anxiety, and stress in Egyptian adults: A cross-sectional study  Naglaa A El-Sherbiny, Asmaa Y Elsary | J Family Community Med. Jan-Apr 2022;29(1):8-16. doi: 10.4103/jfcm.jfcm_290_21. Epub 2022 Jan 19. | Unknown |  |
| 3 | Drug Use in Night Owls May Increase the Risk for Mental Health Problems  Jeevan Fernando, Jan Stochl, Karen D Ersche | Front Neurosci. 2022 Jan 11;15:819566. doi: 10.3389/fnins.2021.819566. eCollection 2021. | Unknown |  |
| 4 | Psychometric properties of Fagerstrom Test of Nicotine Dependence: A systematic review  Manoj Kumar Sharma, L N Suman, Kalpana Srivastava, N Suma, Akash Vishwakarma | Ind Psychiatry J. Jul-Dec 2021;30(2):207-216. doi: 10.4103/ipj.ipj_51_21. Epub 2021 Nov 23. | N/A (Review) |  |
| 5 | Prevalence of tobacco dependence and associated factors among patients with schizophrenia attending their treatments at southwest Ethiopia; hospital-based cross-sectional study  Defaru Desalegn, Zakir Abdu, Mohammedamin Hajure | PLoS One. 2021 Dec 15;16(12):e0261154. doi: 10.1371/journal.pone.0261154. eCollection 2021. | CCA | CCA assumed. If any of the FTND data was missing, it was handled with complete case analysis, but methods not explicitly stated for FTND:  “A total of 524 participants was participating in the study, of which 515 responded.” Response rate refers to other parts of the survey as well (demographics etc.). |
| 6 | Motivation to smoking cessation in head and neck cancer and dysplasia patients in confrontation with the attitudes of otorhinolaryngologists in delivering anti-smoking therapies  Anna Rzepakowska, Bartosz Marcinkiewicz, Michał Żurek, Dominika Wiśniewska, Kazimierz Niemczyk | Eur Arch Otorhinolaryngol. 2021 Dec 10. doi: 10.1007/s00405-021-07209-2. | Complete | Complete assumed. They had FTND scores for each of the 50 patients in their study. |
| 7 | Tobacco smoking and nicotine dependence among patients with respiratory diseases in Vietnam: Status and correlated factors  Phuong Thu Phan, Giap Van Vu, Chau Quy Ngo, Quyen Le Thi Pham, Lan Phuong Thi Doan, Trang Huyen Thi Nguyen, Huong Lan Thi Nguyen, Trang Ha Nguyen, Bach Xuan Tran, Carl A Latkin, Cyrus S H Ho, Roger C M Ho | J Subst Abuse Treat. 2022 Apr;135:108562. doi: 10.1016/j.jsat.2021.108562. Epub 2021 Jul 7. | Complete | Complete assumed. “We approached a total of 550 patients in our study. Finally, 508 patients completed the survey and enrolled in the study.” The FTND survey was only given to the 41 current smokers, and there were FTND scores for all of them. |
| 8 | Smoking and Obstructive Sleep Apnea: Is There An Association between These Cardiometabolic Risk Factors? -Gender Analysis  Despoina Ioannidou, George Kalamaras, Serafeim-Chrysovalantis Kotoulas, Athanasia Pataka | Medicina (Kaunas). 2021 Oct 20;57(11):1137. doi: 10.3390/medicina57111137. | Unknown |  |
| 9 | Exploring the genetic overlap of suicide-related behaviors and substance use disorders  Sarah M C Colbert, Alexander S Hatoum, Andrey Shabalin, Qingqin S Li, Hilary Coon, Elliot C Nelson, Arpana Agrawal, Anna R Docherty, Emma C Johnson | Am J Med Genet B Neuropsychiatr Genet. 2021 Dec;186(8):445-455. doi: 10.1002/ajmg.b.32880. Epub 2021 Nov 25. | Complete | Complete assumed. Their FTND data was taken from Quach, B. C., Bray, M. J., Gaddis, N. C., Liu, M., Palviainen, T., Minica, C. C.,..Hancock, D. B. (2020). Expanding the genetic architecture of nicotine dependence and its shared genetics with multiple traits. From Quach et al. “Beta values and corresponding 95% confidence intervals (CIs) were taken from cross-ancestry meta-analysis of a rs2714700 and b rs1862416 using linear regression models for categorical FTND item responses (1 and 4, closed diamonds) or logistic regression models for binary FTND item responses (2, 3, 5, and 6, open diamonds) across **the iNDiGO studies (N up to 47,569 biologically independent samples with complete FTND data** contributing to the specific item analyses).” |
| 10 | Evaluation of Nicotine Dependence in Adult Population of Eastern Nepal  A Shrestha, V Marla, M Lamsal, D P Sarraf, N Rao | Kathmandu Univ Med J (KUMJ). Apr-Jun 2021;19(74):210-215. | Complete | Complete assumed. Comparing numbers in Tables 2 & 3 to those in text, it appears that they had complete FTND data for all those who smoked or used smokeless tobacco. |
| 11 | Do nicotine dependence influencing and non-influencing behaviors have an association with high nicotine dependence in smokers?  Zhongbo Chen, Xuechan Yu, Dan Lv, Liang Zhang, Hanlu Gao, Younuo Wang 1, Qingwen Su, Hongying Ma 1, Jie Chen, Hui Chen, Qidong Zhuang, Panfeng Fu, Yiming Yu, Zaichun Deng | Tob Induc Dis. 2021 Nov 8;19:86. doi: 10.18332/tid/142866. eCollection 2021. | Complete | Complete assumed. They had FTND scores for all 343 participants in their study. |
| 12 | The relationship of smoking to cg05575921 methylation in blood and saliva DNA samples from several studies  Kelsey Dawes, Allan Andersen, Rachel Reimer, James A Mills, Eric Hoffman, Jeffrey D Long, Shelly Miller, Robert Philibert | Sci Rep. 2021 Nov 3;11(1):21627. doi: 10.1038/s41598-021-01088-7. | N/A (review  of 4 previously published studies) | “To better define this relationship, we conducted a joint analysis of methylation sensitive PCR digital (MSdPCR) assessments of cg05575921 methylation in whole blood and/or saliva DNA to smoking using samples from 421 smokers and 423 biochemically confirmed non-smokers from 4 previously published studies.” |
| 13 | Menthol Smoking and Nicotine Dependence among Black/African American Women Smokers Living in Low-Resource, Rural Communities  Dina M Jones, Margarete C Kulik, Lourdes Baezconde-Garbanati, Sandilyn Bullock, Mignonne C Guy, Pebbles Fagan | Int J Environ Res Public Health  . 2021 Oct 16;18(20):10877. doi: 10.3390/ijerph182010877. | Unknown |  |
| 14 | Betel Quid Dependence Effects on Working Memory and Remote Memory in Chewers with Concurrent Use of Cigarette and Alcohol  Chih-Ying Lee, Wei-Han Wang, Chien-Hung Lee, Ming-Chou Ho | Subst Use Misuse. 2022;57(1):105-113. doi: 10.1080/10826084.2021.1990338. Epub 2021 Oct 22. | Complete | Complete assumed, since the text states that all participants completed the FTND. “The current study recruited 92 male BQ chewers with the concurrent use of cigarettes and alcohol. That is, all participants were current users of BQ, cigarettes and alcohol… All participants completed the Betel Nut Dependency Scale (BNDS) (Li et al., 2012), Fagerstrom Test For Nicotine Dependence (FTND) (Fagerström, 1978; Huang et al., 2006), and Alcohol Use Disorder Identification Test (AUDIT).”  This manuscript needed to be requested from the UA Library. |
| 15 | The effects of preoperative alcohol, tobacco, and psychological stress on postoperative complications: a prospective observational study  Yoshinori Myoga, Haruhiko Manabe, Yoneatsu Osaki | BMC Anesthesiol. 2021 Oct 13;21(1):245. doi: 10.1186/s12871-021-01456-w. | Drop one and exclude if missing item 4 (number of cigarettes smoked per day) | “We assigned zero points to missing items in the AUDIT, FTND, K6, and HADS if more than 75% of all questions had been answered. The questionnaires with the following missing responses were excluded from the analysis: AUDIT responses with items 1–3 missing (questions related to the amount and frequency of drinking), FTND responses with item 4 missing (number of cigarettes smoked per day), and responses with 25% or more missing items (3 or more items in the AUDIT, 2 or more items in the FTND and K6, and 4 or more items in the HADS).” |
| 16 | Is irregular time estimation a common factor in smoking behavior and delay discounting?  C Henrico Stam, Frederik M van der Veen, Ingmar H A Franken | Addict Behav. 2022 Feb;125:107123. doi: 10.1016/j.addbeh.2021.107123. Epub 2021 Sep 21. | Unknown |  |
| 17 | Psychometric investigation of the Chinese version of the Habit, Reward and Fear Scale (HRFS)  Tu Hong, Chuan He, Zhong-Ke Gu, Jun-Jie Xie, Qian Lu, Yong-Qiang Li, Xing-Jun Xu, Ying Shen, Yun-Qiang Wang, Hui Zheng | Brain Behav. 2021 Nov;11(11):e2364. doi: 10.1002/brb3.2364. Epub 2021 Sep 23. | CCA | CCA assumed. “We collected a total of 967 questionnaires during 2019. According to the above screening criteria, the information of 239 non‐smoking participants was screened out, and **28 participants were screened out due to data loss.** Finally, we obtained 700 valid questionnaires.” There were 700 FTND scores: “According to these recommended cut‐off scores, we divided the participants into three groups—those with low dependence (0 to 3 points; n = 430), moderate dependence (3 to 6 points; n = 203), and high dependence (6 to 10 points; n = 67).” |
| 18 | Quality of life of tobacco users- A correlation with duration of use and nicotine dependence  Priyanka Dhawan, Sonu Goel, Abhishek Ghosh | Indian J Tuberc. 2021;68S:S60-S64. doi: 10.1016/j.ijtb.2021.08.025. Epub 2021 Aug 28. | CCA | CCA assumed. “The study initially comprised 140 individuals using tobacco in any form. Ten subjects were excluded because of missing data and other reasons, and thus the study included 130 subjects in total.” |
| 19 | Exploring the role of telehealth: A novel approach to group-based smoking cessation treatment for men incarcerated in a rural state prison  Pamela Valera, Sarah Malarkey, Nadia Smith, Christopher McLaughlin | J Telemed Telecare. 2021 Sep 15;1357633X211034734. doi: 10.1177/1357633X211034734. | CCA | CCA assumed. Table 3 shows sample size n = 20 and that the FTND had 2 missing. (Had to request this paper from UA library.) |
| 20 | A pilot investigation of e-cigarette use and smoking behaviour among patients with chronic airway disease or respiratory symptoms  Hye Seon Kang, Jae Woo Jung, Hye Jung Park, Dong Il Park, Jong Sook Park, Joo Hun Park, Sang Haak Lee, Eun Mi Chun, Jae Yeol Kim, Hye Sook Choi, Korean Smoking Cessation Study Group | Clin Respir J. 2022 Jan;16(1):17-26. doi: 10.1111/crj.13445. Epub 2021 Sep 13. | CCA | CCA assumed. Based on full responses to Question 4 in Table 5 (the only question where all levels of the response were given), only data from 49 participants is included in this FTND score; however, there were 51 participants in the study. |
| 21 | Pictures Library of Smoking Cravings: Development and Verification of Smokers and Non-smokers  Zhongke Gu, Hui Zheng, Zhifei Yin, Huiting Cai, Yongqiang Li, Chunchun Zhao, Yujia Zhai, Kai Xu, Lian Xue, Xingjun Xu, Ying Shen, Ti-Fei Yuan | Front Psychiatry. 2021 Aug 16;12:719782. doi: 10.3389/fpsyt.2021.719782. eCollection 2021. | Complete | Complete assumed. “After this quality check, 816 subjects … finally met the above requirements… Using the Fagerström test of nicotine dependence (FTND), we divided the subjects into non-smokers (n = 211), light smokers (FTND < 5, N = 504), and heavy smokers (FTND ≥ 5, N = 101, as nicotine dependent) (more details are shown in Table 1).” So, there were a total of 816 FTND scores for the 816 subjects. |
| 22 | Tobacco use and nicotine dependence among patients with diabetes and hypertension in Ballabgarh, India  Rakesh Kumar, Shashi Kant, Ankit Chandra, Anand Krishnan | Monaldi Arch Chest Dis. 2021 Aug 10. doi: 10.4081/monaldi.2021.1799. | Complete | Complete assumed. “Table2. Level of nicotine dependence for current predominant smokers (n=60) and current predominant smokeless tobacco users (n=27) among patients with diabetes and hypertension in Ballabgarh, India.” They had FTND scores in their table for 60 current predominant smokers and 27 current predominant smokeless tobacco users. |
| 23 | Leptin Gene and Leptin Receptor Gene Polymorphisms in Alcohol Use Disorder: Findings Related to Psychopathology  Brittney D Browning, Melanie L Schwandt, Mehdi Farokhnia, Sara L Deschaine, Colin A Hodgkinson, Lorenzo Leggio | Front Psychiatry. 2021 Aug 6;12:723059. doi: 10.3389/fpsyt.2021.723059. eCollection 2021. | Unknown |  |
| 24 | Assessment of nicotine dependence among tobacco users visiting outreach programs in Dharan, Nepal: a cross-sectional study  Krishna Subedi, Ashish Shrestha, Tarakant Bhagat | BMC Public Health. 2021 Aug 6;21(1):1515. doi: 10.1186/s12889-021-11535-9. | Complete | Complete assumed. Very similar to reference 10 above. |
| 25 | Is chronic low back pain and radicular neuropathic pain associated with smoking and a higher nicotine dependence? A cross-sectional study using the DN4 and the Fagerström Test for Nicotine Dependence  Emanuel Schembri, Victoria Massalha, Liberato Camilleri, Stephen Lungaro-Mifsud | Agri. 2021 Jul;33(3):155-167. doi: 10.14744/agri.2021.79836. | CCA | “Patients with missing data were excluded from participation in this study.” Also, flow diagram in Fig. 2 shows that 1 person was excluded for incomplete FTND. |
| 26 | Multiethnic Prediction of Nicotine Biomarkers and Association With Nicotine Dependence  Andrew W Bergen, Christopher S McMahan, Stephen McGee, Carolyn M Ervin, Hilary A Tindle, Loïc Le Marchand, Sharon E Murphy, Daniel O Stram, Yesha M Patel, Sungshim L Park, James W Baurley | Nicotine Tob Res. 2021 Nov 5;23(12):2162-2169. doi: 10.1093/ntr/ntab124. | Unknown |  |
| 27 | Comparison between Acupuncture and Nicotine Replacement Therapies for Smoking Cessation Based on Randomized Controlled Trials: A Systematic Review and Bayesian Network Meta-Analysis  Runjing Dai, Yongchun Cao, Hailiang Zhang, Na Zhao, Dong Ren, Xiaomei Jiang, Guisen Zheng, Shisan Bao, Xingke Yan, Jingchun Fan | Evid Based Complement Alternat Med. 2021 Jun 16;2021:9997516. doi: 10.1155/2021/9997516. eCollection 2021. | N/A (Review) |  |
| 28 | Influence of catechol-O-methyltransferase enzyme gene polymorphism on alcohol and tobacco consumption in North Indian treatment seeking population  Rizwana Quraishi, Jaydeep Sharma, Raka Jain, Atul Ambekar | Indian J Psychiatry. May-Jun 2021;63(3):240-244. doi: 10.4103/psychiatry.IndianJPsychiatry_465_20. Epub 2021 Jun 17. | Unknown |  |
| 29 | Validation of the Alcohol Smoking and Substance Involvement Screening Test (ASSIST) in acute psychiatric inpatients  Ana Isabel López-Lazcano, Hugo López-Pelayo, Mercè Balcells-Oliveró, Lidia Segura, Antoni Gual Solé | Adicciones. 2021 Jun 14;0(0):1496. doi: 10.20882/adicciones.1496. | CCA | CCA assumed. Nine participants were removed because they didn’t complete all the tests: Figure 1 “No completaron todos los tests (n = 9).” There were other tests besides the FTND, and no details were given, but CCA seemed to be their approach. |
| 30 | Effects of Oral pH Changes on Smoking Desire  Gökçen Ömeroğlu Şimşek, Gülser Kılınç, Begüm Ergan, Oğuz Kılınç | Balkan Med J. 2021 May;38(3):165-170. doi: 10.5152/balkanmedj.2021.20125. | Unknown |  |
| 31 | More than just statics: Temporal dynamic changes of intrinsic brain activity in cigarette smoking  Mengmeng Wen, Zhengui Yang, Yarui Wei, Huiyu Huang, Ruiping Zheng, Weijian Wang, Xinyu Gao, Mengzhe Zhang, Keke Fang, Yong Zhang, Jingliang Cheng, Shaoqiang Han | Addict Biol. 2021 Nov;26(6):e13050. doi: 10.1111/adb.13050. Epub 2021 Jun 3. | Unknown |  |
| 32 | Alcohol and Tobacco Use in a Tuberculosis Treatment Cohort during South Africa's COVID-19 Sales Bans: A Case Series  Bronwyn Myers, Tara Carney, Jennifer Rooney, Samantha Malatesta, Laura F White, Charles D H Parry, Tara C Bouton, Elizabeth J Ragan, Charles Robert Horsburgh Jr, Robin M Warren, Karen R Jacobson | Int J Environ Res Public Health. 2021 May 19;18(10):5449. doi: 10.3390/ijerph18105449. | Unknown |  |
| 33 | Daily cannabis use in adolescents who smoke tobacco is associated with altered late-stage feedback processing: A high-density electrical mapping study  Kristen P Morie, Jia Wu, Marc N Potenza, Suchitra Krishnan-Sarin, Linda C Mayes, Christopher J Hammond, Michael J Crowley | J Psychiatr Res. 2021 Jul;139:82-90. doi: 10.1016/j.jpsychires.2021.05.022. Epub 2021 May 23. | Unknown |  |
| 34 | Associations between health behaviors and mental health in Australian nursing students  Robert Stanton, Talitha Best, Susan Williams, Corneel Vandelanotte, Christopher Irwin, Penny Heidke, Amornrat Saito, Amanda L Rebar, Trudy Dwyer, Saman Khalesi | Nurse Educ Pract. 2021 May;53:103084. doi: 10.1016/j.nepr.2021.103084. Epub 2021 May 16. | CCA | CCA assumed. “Participants with more than 50% missing data were excluded from  the analysis.” But that applied to more than just the FTND.  “Overall, 500 BN  students (294 CQUniversity and 206 Griffith University) completed the  questionnaire and were included in this study.” Table 2 only has smoking status data (from FTND) for 477 participants. |
| 35 | A qualitative study on attitude towards smoking, quitting and tobacco control policies among current smokers of different socio-economic status  Lalitha Rani Chellappa, Arthi Balasubramaniam, Meignana Arumugham Indiran, Pradeep Kumar Rathinavelu | J Family Med Prim Care. 2021 Mar;10(3):1282-1287. doi: 10.4103/jfmpc.jfmpc_1628_20. Epub 2021 Apr 8. | Unknown |  |
| 36 | No evidence of the clinical utility of single-item breakpoint to inform on tobacco demand in persons with substance use disorders  Alba González-Roz, Roberto Secades-Villa, Gema Aonso-Diego, Sara Weidberg, José R Fernández-Hermida | Psychopharmacology (Berl). 2021 Sep;238(9):2525-2533. doi: 10.1007/s00213-021-05875-y. Epub 2021 May 24. | Unknown |  |
| 37 | Multi-Polygenic Analysis of Nicotine Dependence in Individuals of European Ancestry.  Risner VA, Benca-Bachman CE, Bertin L, Smith AK, Kaprio J, McGeary JE, Chesler E, Knopik VS, Friedman NP, Palmer RHC | Nicotine Tob Res. 2021 Nov 5;23(12):2102-2109. doi: 10.1093/ntr/ntab105. | Complete | Complete assumed. “The second sample comprised 3061 unrelated individuals of European ancestry (51.32% female, mean age: 28.9 [s.d. = 1.70]) with genomic and phenotype data at Wave IV of the National Longitudinal Study of Adolescent to Adult Health (Add Health)… Of the total sample of individuals who endorsed being  current smokers, 50.76% would be considered minimally dependent  (FTND symptom count 0–4, N = 1554), 31.95% would be considered moderately dependent (FTND symptom count 5–6, N = 978),  and 17.28% would be considered highly dependent (FTND symptom  count 7–10, N = 529).” 1554+978+529=3061 |
| 38 | Heaviness of Smoking Index versus Fagerstrom Test for Nicotine Dependence among Current Smokers of Ahmedabad City, India  Parkar Sujal, Patel Anand, Sharma Abhishek | Addict Health. 2021 Jan;13(1):29-35. doi: 10.22122/ahj.v13i1.291. | Complete | Complete assumed. The study had 200 subjects, and Table 1 shows that 113 had low nicotine dependence and 87 had high nicotine dependence based on the FTND score. And 113 + 87 = 200. |
| 39 | The effect of anxiety on nicotine dependence among university students during the COVID-19 pandemic.  Ayran G, Köse S, Küçükoğlu S, Aytekin Özdemir A. | Perspect Psychiatr Care. 2022 Jan;58(1):114-123. doi: 10.1111/ppc.12825. Epub 2021 May 3. | CCA | CCA assumed. “Since the study was collected online, only the participants who completely filled out the questionnaires were included in the study. The study was terminated when a sufficient sample size was reached (n = 503).” |
| 40 | An investigation of smoking habits and mental well-being in healthcare personnel during COVID-19.  Firat M, Demir Gökmen B, Karakurt P. | Perspect Psychiatr Care. 2022 Jan;58(1):108-113. doi: 10.1111/ppc.12819. Epub 2021 Apr 30. | Complete | Complete assumed. FTND score was calculated for the 237 participants who smoked. |
| 41 | Clarifying the Genetic Influences on Nicotine Dependence and Quantity of Use in Cigarette Smokers.  Verhulst B, Clark SL, Chen J, Maes HH, Chen X, Neale MC. | Behav Genet. 2021 Jul;51(4):375-384. doi: 10.1007/s10519-021-10056-w. Epub 2021 Apr 21. | Complete | Complete assumed. “The data for the current study come from four independently collected samples in dbGaP (Tryka et al. 2014). The samples were selected if they had both genotypic and phenotypic information on the same individuals.” |
| 42 | The Development and Assessment of Modified Fagerstrom Test for Nicotine Dependence Scale among Malaysian Single Electronic Cigarette Users.  Rahman AU, Mohamed MHN, Jamshed S, Mahmood S, Iftikhar Baig MA. | J Pharm Bioallied Sci. 2020 Nov;12(Suppl 2):S671-S675. doi: 10.4103/jpbs.JPBS_245_19. Epub 2020 Nov 5. | Unknown |  |
| 43 | Effects of Motivational Interview and Mobile Social Network Support on Smoking Cessation in Male Patients With Coronary Heart Disease.  Chen YH, Wang PC, Ko YL, Wang HL | Hu Li Za Zhi. 2021 Apr;68(2):53-64. doi: 10.6224/JN.202104_68(2).08. | N/A (article in Chinese) |  |
| 44 | Association between functional brain alterations and neuropsychological scales in male chronic smokers using resting-state fMRI.  Weng JC, Huang SY, Lee MS, Ho MC. | Psychopharmacology (Berl). 2021 May;238(5):1387-1399. doi: 10.1007/s00213-021-05819-6. Epub 2021 Mar 27. | Unknown | “The subjects were divided into two groups, namely, 67 chronic smokers and 43nonsmokers as healthy controls…The FTND was administered to assess the severity of nicotine dependence in chronic smokers.” However, I cannot find info on if all the chronic smokers actually completed the FTND. It seems likely all completed it, but it is unknown. |
| 45 | A multicentre tobacco cessation intervention study in the dental setting in Japan.  Nagao T, Fukuta J, Hanioka T, Nakayama Y, Warnakulasuriya S, Sasaki T, Shiota M, Ohno K, Ishigaki Y, Satomura K, Hashimoto S, Goto M, Seto K; Tobacco Cessation Intervention Study for Oral Diseases. | Int Dent J. 2022 Feb;72(1):123-132. doi: 10.1016/j.identj.2021.02.002. Epub 2021 Mar 18. | Complete | Complete assumed. Table 1 shows total sample size of n = 74. Then, from FTND high addiction (n=20) + moderate addiction (n=48) + low addiction (n=6). 20+48+6 = 74. |
| 46 | Predicting smoking and nicotine dependence from the DSM-5 alternative model for personality pathology.  Halberstadt AL, Skrzynski CJ, Wright AGC, Creswell KG. | Personal Disord. 2022 Jan;13(1):84-95. doi: 10.1037/per0000487. Epub 2021 Mar 11. | Complete | Complete assumed. This study had 45.4% = 227/500 current smokers. Table 5 implies that regression for predicting FTND was done on all 227 current smokers. |
| 47 | Alcohol and cigarette smoking consumption as genetic proxies for alcohol misuse and nicotine dependence.  Sanchez-Roige S, Cox NJ, Johnson EO, Hancock DB, Davis LK. | Drug Alcohol Depend. 2021 Apr 1;221:108612. doi: 10.1016/j.drugalcdep.2021.108612. Epub 2021 Feb 15. | Complete | Complete assumed. FTND data was from Quach et al. 2020: “we also included data from a quantitative measure (the Fagerström Test of ND), available only from non-UKB cohorts in the Nicotine Dependence GenOmics (iNDiGO) consortium (total N = 46,213 across 20 cohorts (Quach et al., 2020 )” |
| 48 | A pre-evaluation of psychological factors may positively affect the outcomes of smoking cessation treatments: A comparison in terms of smoking behavior.  Gücük S, Erim BR, Kayhan M. | Tob Prev Cessat. 2021 Feb 12;7:12. doi: 10.18332/tpc/131627. eCollection 2021. | CCA | CCA assumed. No method specifically stated for FTND, but “individuals **excluded** from the study were: those with a severe communication problem that could interfere with responding to the questionnaire**, those who avoided answering some questions** due to their content or who were not able to complete all questions for lack of time (n=122), and those receiving psychiatric treatment for any reason” |
| 49 | Smoking in early adulthood is prospectively associated with prescriptions of antipsychotics, mood stabilizers, antidepressants and anxiolytics.  Rognli EB, Bramness JG, von Soest T. | Psychol Med. 2021 Feb 15;1-10. doi: 10.1017/S0033291720005401. Online ahead of print. | Unknown | The following seems to imply they have complete data for the FTND: “The Fagerström Test for Nicotine Dependence… was included in the questionnaire at T4 to assess nicotine dependence by self-report. The scores range from 0 to 10 (Cronbach’sα= 0.68, based on observations from all respondents who smoked at T4; n= 517).” However, …  “Based on self-reported smoking and the FTND at T4, we divided the material into four categories: Those who had never smoked daily; those who had smoked daily at least once in their lifetime, but not in the last 12 months; those who smoked daily with low dependence (FTDN <4); and those who smoked daily with high dependence (FTDN⩾4) …  Of the total sample of 2602 individuals, 1468 (56.7%) had never been daily smokers, 606 (23.4) were former daily smokers, 305(11.8%) were daily smokers with low dependence and 212(8.2%) were daily smokers with high dependence. Data on smoking was missing for 11 participants.” And we have “Missing data were handled by means of full information maximum likelihood estimation.” It’s not clear if that would apply to missing items on the FTND (probably not – probably just to the total FTND score). |
| 50 | Fagerstrom test for nicotine dependence as an indicator in tobacco-related studies in periodontology.  Salhi L, Seidel L, Albert A, Lambert F. | J Periodontol. 2021 Feb;92(2):298-305. doi: 10.1002/JPER.20-0019. Epub 2020 Aug 16. | Unknown | 34 patients, but unclear if they all had all FTND items answered. |
