# Supplementary material for: Managing missing items in the Fagerström Test for Nicotine Dependence: a simulation study
Source: BMC Med Res Methodol. 2022 May 20;22:145. doi: 10.1186/s12874-022-01637-2 (PMC9121580; doi:10.1186/s12874-022-01637-2)
Supplement: Supplementary file 2 — Additional file 2. R code to clean the ASHLine data and export .rds files for use in Additional file 3 R code. [file 12874_2022_1637_MOESM2_ESM.docx]

Additional File 2: R code to clean the ASHLine data and export .rds files for use in Additional File 3 R code

“Managing missing items in the Fagerström Test for Nicotine Dependence: a simulation study”

Shannon L Gutenkunst & Melanie L Bell

# This R script cleans up the ASHLine data and exports .rds files for use in FTND_sim_ASHline.R (Additional File 3)

# Load libraries ----------------------------------------------------------

# load libraries needed to run this script

# haven package reads SAS (and SPSS and stat) files into R

library(haven) # Used for reading in SAS data from Melanie Bell

library(tidyverse) # Used for wrangling data

# Read in the data --------------------------------------------------------

# Read in the SAS data file using read_sas from the haven package,

# to read data into a tibble, preserve missing values, label columns...

ashline.tb <- read_sas("master4.sas7bdat")

# Clean the data ----------------------------------------------------------

# Rename column name cigs_day_range to af_cigs_day_range, because it will make life easier in the future,

# because now all of the Fagerstrom items start with af_ and are next to each other.

ashline_rename.tb <- ashline.tb %>%

mutate(af_cigs_day_range = cigs_day_range, .keep = "all", .before = af_morning_freq) %>%

dplyr::select(-cigs_day_range)

# Remove those from the dataset who don't have any Fagerstrom questions answered

ashline.clean1.tb <- ashline_rename.tb %>%

filter(if_any(starts_with("af_"), ~!is.na(.x)))

# Calculate the total FTND score and add it to the tibble

ashline.clean2.tb <- ashline.clean1.tb %>%

mutate(FTND = rowSums(dplyr::select(., af_daily_first_use:af_sick_smoke), na.rm = FALSE), .after = af_sick_smoke)

# Note: do NOT use the existing fagerstrom variable for total FTND score variable, b/c it appears wrong about 1% of the time.

# I compared my calc. to it... usually off by one when they differ.

# I am guessing that the call center person just made a mental math mistake ~1% of the time.

compare_FTND_scores.tb <- ashline.clean2.tb %>%

dplyr::select(starts_with("af_") | FTND | fagerstrom) %>%

mutate(compare = near(FTND, fagerstrom)) %>%

filter(compare == FALSE)

# Now create a tibble with just variables that I think might matter (206 is too much!)

ashline.clean3.tb <- ashline.clean2.tb %>%

dplyr::select(intakeid | userid | starts_with("af_") | FTND | clientage | starts_with("smoke") |

ae_work_smoke_others | as_support_others | referral2 | education | gender | hispanic | race |

c_sex_identity | insurance_type | home_language | family_size | intake_date | kids | nrt_before | nrt_request |

perceived_health | starts_with("quit_attempts") | quit_for_24_hours | quit_in_30_days | tob_cigs | tob_start_age | asthma |

hypertension | cancer | copd | diabetes | heartdisease | mental | socialize_smoke | starts_with("policy") | reached | nicotine)

# Further cleaning of the dataset

ashline.clean4.tb <- ashline.clean3.tb %>%

# create a dichotomous variable smoke_allowed_in_home; replies are 0 = no, 1 = yes (either some places or anywhere)

mutate(smoke_allowed_in_home = ifelse((policy_household == 1 | policy_household == 2), 1, 0)) %>%

mutate(smoke_allowed_in_home = ifelse(is.na(policy_household), NA, smoke_allowed_in_home)) %>%

mutate(smoke_allowed_in_home = as.factor(smoke_allowed_in_home)) %>%

# Now ensure variables are the correct data type... they are all doubles above, and need to convert many to factors (some are ordered factors)

mutate(smoke_home_where = ordered(smoke_home_where), smoke_where = ordered(smoke_where), ae_work_smoke_others = ordered(ae_work_smoke_others),

as_support_others = ordered(as_support_others), referral2 = factor(referral2), education = ordered(education), gender=factor(gender), hispanic=factor(hispanic),

race=factor(race), c_sex_identity=factor(c_sex_identity), insurance_type=factor(insurance_type), home_language=factor(home_language), kids=ordered(kids),

nrt_before=factor(nrt_before), nrt_request=factor(nrt_request), perceived_health=ordered(perceived_health), quit_attempts_length=ordered(quit_attempts_length),

quit_for_24_hours=ordered(quit_for_24_hours), quit_in_30_days=factor(quit_in_30_days), tob_cigs=ordered(tob_cigs), socialize_smoke=factor(socialize_smoke),

policy_car=factor(policy_car), policy_household=factor(policy_household), policy_work=factor(policy_work), reached = factor(reached), nicotine = factor(nicotine)) %>%

# remove one 106 year old (the next oldest is 93 years old, so the 106 year old is not representative of usual callers)

filter(clientage < 100) %>%

# NOTE: There are client ages of zero. These should clearly be coded as missing, as I can't imagine a baby calling a smoke quitline...

mutate(clientage = ifelse(clientage == 0, NA, clientage)) %>%

# Create an indicator variable for FTND missingness

mutate(FTND_miss = (is.na(FTND)), .after=FTND) %>%

# The following variables all have levels 0=No, 1=Yes, 2=Don't Know; recode 2 to NA (missing)

mutate(across(c(asthma, cancer, copd, diabetes, heartdisease, hypertension, mental), function(x){ifelse(x == 2, NA, x)})) %>%

# Create an indicator variable for the following question: Does client have a chronic physical health condition?

# (Yes/TRUE/1 if they have at least 1 of the following: Asthma, Cancer, COPD, Diabetes, Heart Disease, Hypertension; No/FALSE/0 otherwise)

mutate(phys_chronic = if_any(c(asthma, cancer, copd, diabetes, heartdisease, hypertension), function(x){x == 1})) %>%

# drop the individual physical chronic conditions

dplyr::select(!c(asthma, cancer, copd, diabetes, heartdisease, hypertension))

# Create a tibble that only includes data from participants who answered ALL of the FTND questions

ashline.clean4.completeFTND.tb <- ashline.clean4.tb %>% filter(FTND_miss == FALSE)

# Export the cleaned data -------------------------------------------------

# Save the cleaned tibbles for analysis in another script

write_rds(ashline.clean4.tb, "ashline.clean4.rds", compress = "none")

write_rds(ashline.clean4.completeFTND.tb, "ashline.clean4.completeFTND.rds", compress = "none")
