# Supplementary material for: Managing missing items in the Fagerström Test for Nicotine Dependence: a simulation study
Source: BMC Med Res Methodol. 2022 May 20;22:145. doi: 10.1186/s12874-022-01637-2 (PMC9121580; doi:10.1186/s12874-022-01637-2)
Supplement: Supplementary file 3 — Additional file 3. R code to simulate data sets and apply different methods. [file 12874_2022_1637_MOESM3_ESM.docx]

Additional File 3: R code to simulate data sets and apply different methods

“Managing missing items in the Fagerström Test for Nicotine Dependence: a simulation study”

Shannon L Gutenkunst & Melanie L Bell

# This R script simulates data sets & applies different methods.

# It starts with the complete cleaned rds file created by the FTND_clean_ASHLine.R (Additional File 2) script.

#--------------------------------------------------------------------------

# Source FTND_sim_functs_ASHLine.R (Additional File 4) for functions & other stuff (incl. libraries)

#--------------------------------------------------------------------------

source('FTND_sim_functs_ASHLine.R')

library(tidymodels) # for regression

# warn=1 prints warnings as they occur

# nwarnings changes the default # of warnings printed from 50 to 10000 here

options(warn = 1, nwarnings = 10000)

#--------------------------------------------------------------------------

# Simulate ----------------------------------------------------------------

#--------------------------------------------------------------------------

# set the seed at the beginning and only once in entire code, for

# reproducible research and so you don't get repeated pseudo-random sequences

set.seed(8)

# Calc. subject-level probabilities for missing FTND from made-up

# logistic regression models for MAR and MNAR and add to tibble.

# The below generate subject-level missingness ofapprox. 0.10, 0.30, and 0.50,

# but the numbers will vary slightly for different random samples.

ash_complete_w_psubs.tb <- ashline.cleaned.completeFTND.tb %>%

# smoke_where: If you smoke at home, where? 0 = No; 1 = Yes, Outside; 3 = Yes, Inside.

# Create indicator vars where smoke_where corresponds to the following (smoke_where1, smoke_where2):

# smoke_where = 0 corresponds to (0, 0), so No becomes the reference level

# smoke_where = 1 corresponds to (1, 0), so smoke_where1 becomes Yes, Outside compared to reference of No

# smoke_where = 3 corresponds to (0, 1), so smoke_where2 becomes Yes, Inside compared to reference of No

# smoke_where = NA corresponds to (NA, NA)

mutate(smoke_where1 = ifelse(smoke_where == 1, 1, 0),

smoke_where2 = ifelse(smoke_where == 3, 1, 0),

smoke_where1 = ifelse(is.na(smoke_where), NA, smoke_where1),

smoke_where2 = ifelse(is.na(smoke_where), NA, smoke_where2)) %>%

psub_mar() %>%

psub_mnar()

run_sim <- function(n_obs, frac_miss_sub, frac_miss_item){

# create the random sample

ash_samp.tb <- ash_complete_w_psubs.tb %>%

# only select variables that are used,

# because don't want to carry too much junk through the simulations

select(intakeid, starts_with("af_"), clientage, gender,

smoke_where1, smoke_where2, smoke_allowed_in_home,

psub_mar.10, psub_mar.30, psub_mar.50,

psub_mnar.10, psub_mnar.30, psub_mnar.50) %>%

# select n_obs random rows from the sample with replacement (replace = TRUE)

slice_sample(n = n_obs, replace = TRUE) %>%

# need row number ID so that no two rows identical, even if same intakeid

# because sample with replacement, and need unique rows for joining

mutate(row_numb = row_number(), .before = intakeid)

#--------------------------------------------------------------------------

# Introduce missingness w/ diff. mechanisms & apply imputation methods ----

#--------------------------------------------------------------------------

# 1. Completely random missingness (MCAR)

amputed_ash_samp_mcar.tb <- mcar(ash_samp.tb, frac_miss_sub = frac_miss_sub, frac_miss_item = frac_miss_item) %>%

apply_impute_methods()

# 2. Missingness based on demographics (MAR)

amputed_ash_samp_mar.tb <- mar(ash_samp.tb, frac_miss_sub = frac_miss_sub, frac_miss_item = frac_miss_item) %>%

apply_impute_methods()

# 3. Missingness based on value of FTND (MNAR)

amputed_ash_samp_mnar.tb <- mnar(ash_samp.tb, frac_miss_sub = frac_miss_sub, frac_miss_item = frac_miss_item) %>%

apply_impute_methods()

#--------------------------------------------------------------------------

# Calculate bias & precision at individual & population levels ------------

#--------------------------------------------------------------------------

# Combine simulated datasets for different missingness mechanisms into one.

# First drop the unnecessary columns in each tibble.

mcar_sim.tb <- amputed_ash_samp_mcar.tb %>%

select(row_numb, intakeid, starts_with("FTND_"))

mar_sim.tb <- amputed_ash_samp_mar.tb %>%

select(row_numb, intakeid, starts_with("FTND_"))

mnar_sim.tb <- amputed_ash_samp_mnar.tb %>%

select(row_numb, intakeid, starts_with("FTND_"))

# Match tibbles based on row_numb

sim.tb <- full_join(mcar_sim.tb, mar_sim.tb, by = c("row_numb", "intakeid")) %>%

full_join(., mnar_sim.tb, by = c("row_numb", "intakeid"))

# Original cleaned complete dataset "gold standard"

ash_gold.tb <- ashline.cleaned.completeFTND.tb %>%

mutate(FTNDgold = FTND) %>%

select(intakeid, FTNDgold, gender, reached, smoke_allowed_in_home)

# First match sim.tb & ash_gold.tb based on intakeid

calc.tb <- left_join(sim.tb, ash_gold.tb, by = "intakeid")

# Calculate bias & (im)precision for the popl'n mean & bias for popl'n sd

# First calc. mean & sd of the observed (complete) total FTND score

mean_FTNDgold <- mean(calc.tb$FTNDgold)

# sd_FTNDgold <- sd(calc.tb$FTNDgold) # not the correct thing to compare against

# calculate regression coefficient for the complete dataset for FTNDgold ~ smoke_allowed_in_home

regcoeff_gold <- tidy(summary(lm(calc.tb$FTNDgold ~ calc.tb$smoke_allowed_in_home)))$estimate[[2]]

# regcoeffse_gold <- tidy(summary(lm(calc.tb$FTNDgold ~ calc.tb$smoke_allowed_in_home)))$std.error[[2]] # not correct to compare against

pop_summary.tb <- calc.tb %>%

# calc. bias as diff. between mean of imputed and mean of observed (complete) total FTND score;

# calc. mean & SE of FTND for use in calculating bias of SE of FTND later

# calc. N = total number of FTND scores on which the result is based

# calc. bias of regression coeff. for regression FTND ~ smoke_allowed_in_home

# calc. regression coeff. and its SE for use in calculating bias of SE of regression coeff. later

summarize(across(starts_with("FTND_"), list(pop_meanbias = function(x){mean(x, na.rm = TRUE) - mean_FTNDgold},

pop_meanbiasperc = function(x){(mean(x, na.rm = TRUE) - mean_FTNDgold)/mean_FTNDgold*100.},

# pop_sdbias = function(x){sd(x, na.rm = TRUE) - sd_FTNDgold},

# pop_sdbiasperc = function(x){(sd(x, na.rm = TRUE) - sd_FTNDgold)/sd_FTNDgold*100.},

pop_mean = function(x){mean(x, na.rm = TRUE)}, # retain for calculating bias of SE of FTND later

pop_meanse = function(x){sd(x, na.rm = TRUE)/sqrt(sum(!is.na(x)))}, # retain for calculating bias of SE of FTND later

pop_deltaN = function(x){length(x) - sum(!is.na(x))},

pop_deltaNperc = function(x){(length(x) - sum(!is.na(x)))/length(x)*100.},

# calculate bias of regression coefficient for FTNDgold ~ smoke_allowed_in_home for each method & miss. mech.

pop_regrcoeffbias = function(x){tidy(summary(lm(x ~ smoke_allowed_in_home, data = calc.tb)))$estimate[[2]] - regcoeff_gold},

pop_regrcoeffbiasperc = function(x){(tidy(summary(lm(x ~ smoke_allowed_in_home, data = calc.tb)))$estimate[[2]] - regcoeff_gold)/regcoeff_gold*100.},

# pop_regrcoeffsebias = function(x){tidy(summary(lm(x ~ smoke_allowed_in_home, data = calc.tb)))$std.error[[2]] - regcoeffse_gold},

# pop_regrcoeffsebiasperc = function(x){(tidy(summary(lm(x ~ smoke_allowed_in_home, data = calc.tb)))$std.error[[2]] - regcoeffse_gold)/regcoeffse_gold*100.}

pop_regrcoeffmean = function(x){tidy(summary(lm(x ~ smoke_allowed_in_home, data = calc.tb)))$estimate[[2]]},

pop_regrcoeffse = function(x){tidy(summary(lm(x ~ smoke_allowed_in_home, data = calc.tb)))$std.error[[2]]}

)))

# # Calculate bias & precision for individuals

# # First calc. bias as the diff. between the individual‚Äôs imputed & their observed (complete) total FTND score;

# # then calc precision as that difference squared for each individual

# calc.tb <- calc.tb %>%

# mutate(across(starts_with("FTND_"), list(indiv_meanbias = function(x){x - FTNDgold},

# indiv_meanprec = function(x){(x - FTNDgold)^2})))

# indiv_summary.tb <- calc.tb %>%

# # Finally, calculate the average of the individual bias and precision

# summarize(across(matches("indiv"), ~ mean(.x, na.rm = TRUE)))

#

# # All of the output parameters we care about will be 1 row in a larger tibble

# output_1_run.tb <- tibble(pop_summary.tb, indiv_summary.tb)

output_1_run.tb <- pop_summary.tb

}

# number of repetitions of simulation: nrep

# number of observations to randomly sample: n_obs (common to vary this in simulation studies)

# probability of a subject having missing data: frac_miss_sub

# probability of missing item if subject is eligible for missingness: frac_miss_item

# XXX: For debugging

# nrep <- 1000

# n_obs_vector <- c(788)

# frac_miss_sub_vector <- c(0.50)

# frac_miss_item_vector <- c(0.10, 0.70)

nrep <- 1000

n_obs_vector <- c(52, 788)

frac_miss_sub_vector <- c(0.10, 0.30, 0.50)

frac_miss_item_vector <- c(0.10, 0.30, 0.50, 0.70)

for (i in seq_along(n_obs_vector)){

for (j in seq_along(frac_miss_sub_vector)){

for (k in seq_along(frac_miss_item_vector)){

# print nobs, psub, and pitem values

print(paste0("nobs = ", n_obs_vector[i], ", psub = ", frac_miss_sub_vector[j], ", pitem = ", frac_miss_item_vector[k]))

# name of data frame to write simulation results to

fname <- paste0("sim_results/nrep_", nrep,

"_nobs_", n_obs_vector[i],

"_fracmisssub_", frac_miss_sub_vector[j],

"_fracmissitem_", frac_miss_item_vector[k], "_.rds" )

# write simulation results to the data frame

m.df <- map_dfr(1:nrep, ~run_sim(n_obs = n_obs_vector[i],

frac_miss_sub = frac_miss_sub_vector[j],

frac_miss_item = frac_miss_item_vector[k]))

# Now that we have the data from our 1,000 runs, we can calculate the empirical SE_method = SD(1,000 mean FTND scores for that method)

# and compare against that for a measure of precision.

# Also similarly calc. the empirical SE of the regr. coeff. as SD(1,000 b's for that method) and compare against that.

# add the gold standard of empirical SE to the dataset for comparison

m.df <- m.df %>%

mutate(across(ends_with('mean'), list(segold = function(x){sd(x, na.rm = TRUE)}

)))

# use for loops to do the comparisons, looping over combinations of miss_mech and method

miss_mech_name <- c("mcar", "mar", "mnar")

method_name <- c("cca", "dropone", "itemmean", "itemmeanhr", "proration", "hotdeck")

for (ii in miss_mech_name){

for (jj in method_name){

# Calc. bias of SE of mean FTND

col1 <- paste0("FTND_", ii, "_", jj, "_pop_meanse")

col2 <- paste0("FTND_", ii, "_", jj, "_pop_mean_segold")

col2newname <- paste0("FTND_", ii, "_", jj, "_pop_meansegold")

col3 <- paste0("FTND_", ii, "_", jj, "_pop_sebias")

col4 <- paste0("FTND_", ii, "_", jj, "_pop_sebiasperc")

m.df[col3] <- m.df[col1] - m.df[col2]

m.df[col4] <- (m.df[col1] - m.df[col2])/m.df[col2]*100.

# Calc. bias of SE of regr. coeff.

col5 <- paste0("FTND_", ii, "_", jj, "_pop_regrcoeffse")

col6 <- paste0("FTND_", ii, "_", jj, "_pop_regrcoeffmean_segold")

col6newname <- paste0("FTND_", ii, "_", jj, "_pop_regrcoeffmeansegold")

col7 <- paste0("FTND_", ii, "_", jj, "_pop_regrcoeffsebias")

col8 <- paste0("FTND_", ii, "_", jj, "_pop_regrcoeffsebiasperc")

m.df[col7] <- m.df[col5] - m.df[col6]

m.df[col8] <- (m.df[col5] - m.df[col6])/m.df[col6]*100.

# rename the empirical SEs so they don't have an underscore

# (the underscore creates a problem for the analysis program FTND_analyze_sim_results)

m.df <- rename(m.df, !!col2newname := {{col2}}, !!col6newname := {{col6}})

}

}

# save each data frame for analysis in another script

write_rds(m.df, fname, compress = "bz2")

}

}

}

# revert to default of 50 warning messages; keep warn = 1 so warnings are printed as they occur

options(warn = 1, nwarnings = 50)
