# Supplementary material for: Managing missing items in the Fagerström Test for Nicotine Dependence: a simulation study
Source: BMC Med Res Methodol. 2022 May 20;22:145. doi: 10.1186/s12874-022-01637-2 (PMC9121580; doi:10.1186/s12874-022-01637-2)
Supplement: Supplementary file 4 — Additional file 4. R code with functions to use in the simulation, called from Additional file 3. [file 12874_2022_1637_MOESM4_ESM.docx]

Additional File 4: R code with functions to use in the simulation, called from Additional File 3

“Managing missing items in the Fagerström Test for Nicotine Dependence: a simulation study”

Shannon L Gutenkunst & Melanie L Bell

# This is where I put many of the functions to use in the simulation,

# to make the simulation part cleaner and easier to follow. They are called

# from FTND_sim_ASHLine.R (Additional File 3).

#--------------------------------------------------------------------------

# Load libraries ----------------------------------------------------------

#--------------------------------------------------------------------------

library(tidyverse) # used for data wrangling

library(simputation) # used for hotdeck imputation method

#--------------------------------------------------------------------------

# Read in the data & create vector of FTND_item_colnames-------------------

#--------------------------------------------------------------------------

# Read the cleaned rds files output by FTND_clean_ASHLine.R into tibbles for analysis

ashline.cleaned.tb <- read_rds("ashline.clean4.rds")

ashline.cleaned.completeFTND.tb <- read_rds("ashline.clean4.completeFTND.rds")

# Create a vector of the column names of the individual FTND items

FTND_item_colnames <- ashline.cleaned.tb %>% select(starts_with("af_")) %>% colnames()

#--------------------------------------------------------------------------

# Make up logistic regression models for MAR and MNAR & calc. subj. probs.

#--------------------------------------------------------------------------

# add subject-level probabilities from a made up logistic model for MAR

# logit(psub) = ln(psub/(1-psub)) = b0 + b1*gender + b2*smoke_where1 + b3*smoke_where2

psub_mar <- function(tb){

# choose b0 empirically to set the desired subject-level missingness of 0.10, 0.30, or 0.50.

# b0 = -0.47 corresponds to frac_sub_missing = 0.10 for b1 = 0.20, b2 = -2.12, b3 = -1.99

# b0 = 0.91 corresponds to frac_sub_missing = 0.30 for b1 = 0.20, b2 = -2.12, b3 = -1.99

# b0 = 1.77 corresponds to frac_sub_missing = 0.50 for b1 = 0.20, b2 = -2.12, b3 = -1.99

# b0 <- c(31.70, 34.21, 35.81) # these are coeff. for 20*(b1 = 0.20, b2 = -2.12, b3 = -1.99)

b0 <- c(-0.47, 0.91, 1.77)

b1 <- 0.20 # males are 1.22 times more likely to have missing than females.

b2 <- -2.12 # those who smoke at home outside are 0.12 times as likely to have missing than those who don't smoke at home.

b3 <- -1.99 # those who smoke at home inside are 0.14 times as likely to have missing than those who don't smoke at home.

# solve logistic regression model for each level of p_sub and add to tibble

# replace NAs for p_sub (because of missing gender or smoke_where) with mean of p_sub

tb <- tb %>%

mutate(psub_mar.10 = exp(b0[1] + b1*as.numeric(gender) + b2*smoke_where1 + b3*smoke_where2)/(1+exp(b0[1] + b1*as.numeric(gender) + b2*smoke_where1 + b3*smoke_where2))) %>%

mutate(psub_mar.10 = replace_na(psub_mar.10, mean(psub_mar.10, na.rm = TRUE))) %>%

mutate(psub_mar.30 = exp(b0[2] + b1*as.numeric(gender) + b2*smoke_where1 + b3*smoke_where2)/(1+exp(b0[2] + b1*as.numeric(gender) + b2*smoke_where1 + b3*smoke_where2))) %>%

mutate(psub_mar.30 = replace_na(psub_mar.30, mean(psub_mar.30, na.rm = TRUE))) %>%

mutate(psub_mar.50 = exp(b0[3] + b1*as.numeric(gender) + b2*smoke_where1 + b3*smoke_where2)/(1+exp(b0[3] + b1*as.numeric(gender) + b2*smoke_where1 + b3*smoke_where2))) %>%

mutate(psub_mar.50 = replace_na(psub_mar.50, mean(psub_mar.50, na.rm = TRUE)))

tb

}

# add subject-level probabilities from a made up logistic model for MNAR

# logit(psub) = ln(psub/(1-psub)) = b0 + b1*FTND

psub_mnar <- function(tb){

# choose b0 empirically to set the desired subject-level missingness of 0.10, 0.30, or 0.50.

# b0 = -3.22 corresponds to frac_sub_missing = 0.10 for b1 = 0.20

# b0 = -1.84 corresponds to frac_sub_missing = 0.30 for b1 = 0.20

# b0 = -0.93 corresponds to frac_sub_missing = 0.50 for b1 = 0.20

b0 <- c(-3.22, -1.84, -0.93)

b1 <- 0.2 # corresponding to OR 1.22 for a 1-point increase in FTND

# solve logistic regression model for each level of p_sub and add to tibble

# No missing FTND in complete data, so no need to replace NAs for p_sub

tb <- tb %>%

mutate(psub_mnar.10 = exp(b0[1] + b1*as.numeric(FTND))/(1+exp(b0[1] + b1*as.numeric(FTND)))) %>%

mutate(psub_mnar.30 = exp(b0[2] + b1*as.numeric(FTND))/(1+exp(b0[2] + b1*as.numeric(FTND)))) %>%

mutate(psub_mnar.50 = exp(b0[3] + b1*as.numeric(FTND))/(1+exp(b0[3] + b1*as.numeric(FTND))))

tb

}

# checking that desired subject-level missingness achieved

# ash_complete_w_psubs.tb <- ashline.cleaned.completeFTND.tb %>%

# mutate(smoke_where1 = ifelse(smoke_where == 1, 1, 0),

# smoke_where2 = ifelse(smoke_where == 3, 1, 0),

# smoke_where1 = ifelse(is.na(smoke_where), NA, smoke_where1),

# smoke_where2 = ifelse(is.na(smoke_where), NA, smoke_where2)) %>%

# psub_mar() %>%

# # psub_mnar() %>%

# mutate(missing_sub_mar.10 = rbinom(n(), 1, psub_mar.10)) %>%

# mutate(missing_sub_mar.30 = rbinom(n(), 1, psub_mar.30)) %>%

# mutate(missing_sub_mar.50 = rbinom(n(), 1, psub_mar.50)) # %>%

# # mutate(missing_sub_mnar.10 = rbinom(n(), 1, psub_mnar.10)) %>%

# # mutate(missing_sub_mnar.30 = rbinom(n(), 1, psub_mnar.30)) %>%

# # mutate(missing_sub_mnar.50 = rbinom(n(), 1, psub_mnar.50))

# ash_complete_w_psubs.tb %>% filter(missing_sub_mar.10 == 1) %>% count()/nrow(ash_complete_w_psubs.tb)

# ash_complete_w_psubs.tb %>% filter(missing_sub_mar.30 == 1) %>% count()/nrow(ash_complete_w_psubs.tb)

# ash_complete_w_psubs.tb %>% filter(missing_sub_mar.50 == 1) %>% count()/nrow(ash_complete_w_psubs.tb)

# # ash_complete_w_psubs.tb %>% filter(missing_sub_mnar.10 == 1) %>% count()/nrow(ash_complete_w_psubs.tb)

# # ash_complete_w_psubs.tb %>% filter(missing_sub_mnar.30 == 1) %>% count()/nrow(ash_complete_w_psubs.tb)

# # ash_complete_w_psubs.tb %>% filter(missing_sub_mnar.50 == 1) %>% count()/nrow(ash_complete_w_psubs.tb)

#--------------------------------------------------------------------------

# Calculate the number of FTND items not missing & add to tibble ----------

#--------------------------------------------------------------------------

# Calculate the number of FTND items not missing & add to tibble,

# because will need it in several methods below

add_n_FTND_items_no_miss <- function(tb){

tb <- tb %>%

mutate(n_FTND_items_no_miss = rowSums(!is.na(select(., af_daily_first_use:af_sick_smoke))), .after = af_sick_smoke)

}

#--------------------------------------------------------------------------

# Missingness generation mechanism functions ------------------------------

#--------------------------------------------------------------------------

# MCAR --------------------------------------------------------------------

mcar <- function(tb, frac_miss_sub = frac_miss_sub, frac_miss_item = frac_miss_item){

# add missing mechanism attribute for use later in variable naming

attr(tb, 'missing_mech') <- 'mcar'

# choose random sujects eligible for missingness based on frac_miss_sub

miss_sub_indic.tb <- tb %>% mutate(missing_sub = rbinom(n(), 1, frac_miss_sub))

# subjects eligible for missingness

miss_sub.tb <- miss_sub_indic.tb %>% filter(missing_sub == 1)

# subjects not eligible for missingess

nonmiss_sub.tb <- miss_sub_indic.tb %>% filter(missing_sub == 0)

# Generate missingingness pattern for FTND items data for subjects eligible for missingness.

dimensions <- miss_sub.tb %>% select(starts_with("af_")) %>% dim()

missing_mask <- rbinom(dimensions[1]*dimensions[2], 1, frac_miss_item) %>%

as.logical() %>%

matrix(ncol=dimensions[2], nrow=dimensions[1])

# Apply missingness pattern to copy of FTND items data

amputed_ash_samp_af_data.tb <- miss_sub.tb %>% select(starts_with("af_"))

amputed_ash_samp_af_data.tb[missing_mask] <- NA

# Replace FTND items data with amputed values in copy of original tibble

amputed_miss_sub.tb <- miss_sub.tb

amputed_miss_sub.tb[FTND_item_colnames] <- amputed_ash_samp_af_data.tb

# Recombine the sample of subjects allowed to have amputed items with the rest

# of the sample (with no amputed items), and return the tibble with amputed values.

bind_rows(amputed_miss_sub.tb, nonmiss_sub.tb) %>%

arrange(row_numb) %>% # put back in the original order the sample was drawn

add_n_FTND_items_no_miss() # add number of FTND items not missing to tibble

}

# MAR ---------------------------------------------------------------------

mar <- function(tb, frac_miss_sub = frac_miss_sub, frac_miss_item = frac_miss_item){

# add missing mechanism attribute for use later in variable naming

attr(tb, 'missing_mech') <- 'mar'

# choose sujects for missingness based on their probs. from made-up logistic regr.

if (frac_miss_sub == 0.10){

miss_sub_indic.tb <- tb %>% mutate(missing_sub = rbinom(n(), 1, psub_mar.10))

} else if (frac_miss_sub == 0.30){

miss_sub_indic.tb <- tb %>% mutate(missing_sub = rbinom(n(), 1, psub_mar.30))

} else if (frac_miss_sub == 0.50){

miss_sub_indic.tb <- tb %>% mutate(missing_sub = rbinom(n(), 1, psub_mar.50))

} else {print("Error: not allowed fraction of missing subjects")}

# subjects eligible for missingness

miss_sub.tb <- miss_sub_indic.tb %>% filter(missing_sub == 1)

# subjects not eligible for missingess

nonmiss_sub.tb <- miss_sub_indic.tb %>% filter(missing_sub == 0)

# Generate missingingness pattern for FTND items data for subjects eligible for missingness.

dimensions <- miss_sub.tb %>% select(starts_with("af_")) %>% dim()

missing_mask <- rbinom(dimensions[1]*dimensions[2], 1, frac_miss_item) %>%

as.logical() %>%

matrix(ncol=dimensions[2], nrow=dimensions[1])

# Apply missingness pattern to copy of FTND items data

amputed_ash_samp_af_data.tb <- miss_sub.tb %>% select(starts_with("af_"))

amputed_ash_samp_af_data.tb[missing_mask] <- NA

# Replace FTND items data with amputed values in copy of original tibble

amputed_miss_sub.tb <- miss_sub.tb

amputed_miss_sub.tb[FTND_item_colnames] <- amputed_ash_samp_af_data.tb

# Recombine the sample of subjects allowed to have amputed items with the rest

# of the sample (with no amputed items), and return the tibble with amputed values.

bind_rows(amputed_miss_sub.tb, nonmiss_sub.tb) %>%

arrange(row_numb) %>% # put back in the original order the sample was drawn

add_n_FTND_items_no_miss() # add number of FTND items not missing to tibble

}

# MNAR --------------------------------------------------------------------

mnar <- function(tb, frac_miss_sub = frac_miss_sub, frac_miss_item = frac_miss_item){

# add missing mechanism attribute for use later in variable naming

attr(tb, 'missing_mech') <- 'mnar'

# choose sujects for missingness based on their probs. from made-up logistic regr.

if (frac_miss_sub == 0.10){

miss_sub_indic.tb <- tb %>% mutate(missing_sub = rbinom(n(), 1, psub_mnar.10))

} else if (frac_miss_sub == 0.30){

miss_sub_indic.tb <- tb %>% mutate(missing_sub = rbinom(n(), 1, psub_mnar.30))

} else if (frac_miss_sub == 0.50){

miss_sub_indic.tb <- tb %>% mutate(missing_sub = rbinom(n(), 1, psub_mnar.50))

} else {print("Error: not allowed fraction of missing subjects")}

# subjects eligible for missingness

miss_sub.tb <- miss_sub_indic.tb %>% filter(missing_sub == 1)

# subjects not eligible for missingess

nonmiss_sub.tb <- miss_sub_indic.tb %>% filter(missing_sub == 0)

# Generate missingingness pattern for FTND items data for subjects eligible for missingness.

dimensions <- miss_sub.tb %>% select(starts_with("af_")) %>% dim()

missing_mask <- rbinom(dimensions[1]*dimensions[2], 1, frac_miss_item) %>%

as.logical() %>%

matrix(ncol=dimensions[2], nrow=dimensions[1])

# Apply missingness pattern to copy of FTND items data

amputed_ash_samp_af_data.tb <- miss_sub.tb %>% select(starts_with("af_"))

amputed_ash_samp_af_data.tb[missing_mask] <- NA

# Replace FTND items data with amputed values in copy of original tibble

amputed_miss_sub.tb <- miss_sub.tb

amputed_miss_sub.tb[FTND_item_colnames] <- amputed_ash_samp_af_data.tb

# Recombine the sample of subjects allowed to have amputed items with the rest

# of the sample (with no amputed items), and return the tibble with amputed values.

bind_rows(amputed_miss_sub.tb, nonmiss_sub.tb) %>%

arrange(row_numb) %>% # put back in the original order the sample was drawn

add_n_FTND_items_no_miss() # add number of FTND items not missing to tibble

}

#--------------------------------------------------------------------------

# Methods functions -------------------------------------------------------

#--------------------------------------------------------------------------

# Create FTND variable names for diff. missingness mech. & methods --------

make_FTND_var_name <- function(missing_mech, method = ""){

if (method == "") {

return("unknown_method")

}

paste0("FTND", "_", missing_mech, "_", method)

}

# Complete Case Analysis (CCA) --------------------------------------------

# If participants have any item(s) missing, their total FTND score is also missing.

cca <- function(tb){

missing_mech <- attr(tb, 'missing_mech')

varname <- make_FTND_var_name(missing_mech, method = "cca")

tb %>%

mutate(!!varname := rowSums(select(., af_daily_first_use:af_sick_smoke), na.rm = FALSE), .after = n_FTND_items_no_miss)

}

# Drop One ----------------------------------------------------------------

# If at most 1 item is missing, calc. FTND total score without it

# (i.e., the missing item is assumed to have a score of 0).

# If >1 item is missing, code the FTND total score as missing.

drop_one <- function(tb){

missing_mech <- attr(tb, 'missing_mech')

varname <- make_FTND_var_name(missing_mech, method = "dropone")

tb %>%

mutate(!!varname := ifelse(n_FTND_items_no_miss >= 5, rowSums(select(., af_daily_first_use:af_sick_smoke), na.rm = TRUE), NA), .after = n_FTND_items_no_miss)

}

# Item Mean ---------------------------------------------------------------

# Replace each missing value with the item mean across all subjects,

# then calculate total FTND

item_mean <- function(tb){

missing_mech <- attr(tb, 'missing_mech')

varname <- make_FTND_var_name(missing_mech, method = "itemmean")

tb_calc <- tb %>%

mutate(across(c(starts_with("af_")), function(x) replace(x, is.na(x), mean(x, na.rm = TRUE)))) %>%

mutate(FTND_item_mean = rowSums(select(., af_daily_first_use:af_sick_smoke), na.rm = FALSE), .after = n_FTND_items_no_miss)

tb %>%

mutate(!!varname := tb_calc$FTND_item_mean, .after = n_FTND_items_no_miss)

}

# Item Mean with Half Rule ------------------------------------------------

# Replace each missing value with the item mean across all subjects,

# ONLY IF at least half (3) of the items for a subject are not missing.

# Then calculate total FTND.

item_mean_half_rule <- function(tb){

missing_mech <- attr(tb, 'missing_mech')

varname <- make_FTND_var_name(missing_mech, method = "itemmeanhr")

tb_calc <- tb %>%

mutate(across(c(starts_with("af_")), function(x){ifelse(n_FTND_items_no_miss >= 3, replace(x, is.na(x), mean(x, na.rm = TRUE)), x)})) %>%

mutate(FTND_item_mean_hr = rowSums(select(., af_daily_first_use:af_sick_smoke), na.rm = FALSE), .after = n_FTND_items_no_miss)

tb %>%

mutate(!!varname := tb_calc$FTND_item_mean_hr, .after = n_FTND_items_no_miss)

}

# Proration ---------------------------------------------------------------

# If at least half of items on FTND are non-missing, then use proration

# (normalized, or weighted mean, or whatever you want to call it, because

# the six FTND items have different ranges); else code as missing

proration <- function(tb){

missing_mech <- attr(tb, 'missing_mech')

varname <- make_FTND_var_name(missing_mech, method = "proration")

# Two items (af_daily_first_use & af_cigs_day_range; the 1st and 4th listed) have possible points (0, 1, 2, 3);

# the other four items all have possible points (0, 1). The weight vector below helps account for that.

weight_vector <- c(3, 1, 1, 3, 1, 1)

names(weight_vector) <- FTND_item_colnames

prorate.tb <- tb %>%

# want sums over rows... rowSums didn't seem to work right with the vector multiplication

rowwise() %>%

# calculate the total possible score of the questions the client actually answered

mutate(tot_poss = (sum(weight_vector * !is.na(c_across(af_daily_first_use:af_sick_smoke)), na.rm = FALSE)),

# calculate the total score of the questions the client actually answered

tot_of_ans = sum(c_across(af_daily_first_use:af_sick_smoke), na.rm = TRUE),

# calculate what I am calling the client fraction (originally called weight, but that's confusing)

# as the ratio of the two numbers above; it will be a number in the range of [0,1].

wt_for_client = tot_of_ans / tot_poss, .after = n_FTND_items_no_miss) %>%

ungroup()

# Replacing NAs for each client (= each row) using client fraction times the weight for

# the item (i.e, 3 times the weight for the client for af_daily_first_use & af_cigs_day_range;

# 1 times the weight for the client for the rest of the items),

# if at least half (3) of items on FTND are non-missing.

# Replacing NAs like this across rows seems trickier than replacing them down columns. I followed

# https://stackoverflow.com/questions/63623026/replace-nas-with-row-minimum-for-selected-columns

prorate2.tb <-prorate.tb %>%

select(starts_with("af_"), row_numb, wt_for_client) %>%

pivot_longer(cols = -c(row_numb, wt_for_client)) %>%

group_by(row_numb) %>%

mutate(replace_Val = wt_for_client, Flag = sum(is.na(value))) %>%

ungroup() %>%

mutate(value = ifelse(is.na(value) & Flag <= 3 & name %in% c("af_daily_first_use", "af_cigs_day_range"), 3*replace_Val, value)) %>%

mutate(value = ifelse(is.na(value) & Flag <= 3, replace_Val, value)) %>%

select(-c(replace_Val, Flag)) %>%

pivot_wider(names_from = name, values_from = value)

prorate.tb[FTND_item_colnames] <- prorate2.tb[FTND_item_colnames]

# use the imputed values for the individual items to estimate the total FTND score for each client

prorate.tb <- prorate.tb %>%

mutate(FTND_proration = rowSums(select(., af_daily_first_use:af_sick_smoke), na.rm = FALSE), .after = n_FTND_items_no_miss)

tb %>% mutate(!!varname := prorate.tb$FTND_proration, .after = n_FTND_items_no_miss)

}

# Hot Deck Imputation -----------------------------------------------------

# Below I use k-nearest neighbors hotdeck imputation, which for each row with

# one or more missing FTND item scores (a recipient row), first uses the predictors

# to calculate Gower's distance between rows to determine the k most similar

# rows where those items are not missing, and second chooses a donor from those k

# rows to donate imputed values to the recipient row.

# Gower's distance reference: Gower, J.C., 1971. A general coefficient of similarity

# and some of its properties. Biometrics, pp.857‚Äì871.

# k = 5 is default

# For pool = "multivariate", a pool of donors is generated for each pattern of missing data,

# and if a row has multiple missing items, all imputations are taken from a single donor.

# impute_knn(data, imputed_variables ~ distance_variables)

hotdeck <- function(tb){

missing_mech <- attr(tb, 'missing_mech')

varname <- make_FTND_var_name(missing_mech, method = "hotdeck")

fmla <- as.formula(paste(paste(FTND_item_colnames, collapse=" + "), "~ gender + smoke_allowed_in_home + ", paste(paste(FTND_item_colnames, collapse=" + "))))

hotdeck.tb <- as.data.frame(tb) %>% # impute_knn not working with tibble

impute_knn(fmla, k=5, pool = "multivariate") %>%

as_tibble() %>%

mutate(FTND_hotdeck = rowSums(select(., af_daily_first_use:af_sick_smoke), na.rm = FALSE), .after = n_FTND_items_no_miss)

tb %>% mutate(!!varname := hotdeck.tb$FTND_hotdeck, .after = n_FTND_items_no_miss)

}

#--------------------------------------------------------------------------

# Apply all different methods for estimating total FTND score -------------

#--------------------------------------------------------------------------

apply_impute_methods <- function(df){

df %>%

cca() %>%

drop_one() %>%

item_mean() %>%

item_mean_half_rule() %>%

proration() %>%

hotdeck()

}
