# Supplementary material for: Managing missing items in the Fagerström Test for Nicotine Dependence: a simulation study
Source: BMC Med Res Methodol. 2022 May 20;22:145. doi: 10.1186/s12874-022-01637-2 (PMC9121580; doi:10.1186/s12874-022-01637-2)
Supplement: Supplementary file 5 — Additional file 5. Rmarkdown code for plots of FTND simulation results plotted in Additional file 6. [file 12874_2022_1637_MOESM5_ESM.docx]

Additional File 5: Rmarkdown code for plots of FTND simulation results plotted in Additional File 6

“Managing missing items in the Fagerström Test for Nicotine Dependence: a simulation study”

Shannon L Gutenkunst & Melanie L Bell

---

title: "Additional File 6: Plots of FTND simulation results"

subtitle: '"A simulation study analyzing how to manage missing items in the Fagerstr√∂m Test for Nicotine Dependence"'

author: "Shannon L Gutenkunst & Melanie L Bell"

# date: "December 1, 2021"

output:

pdf_document: default

html_document: default

word_document: default

---

```{r setup, include=FALSE}

knitr::opts_chunk$set(

echo = FALSE,

message = FALSE,

warning = FALSE

)

```

There is a separate figure for each combination of sample size ($n_{obs}$ = 52 and 788) and probability of subject-level missingness ($p_{sub}$ = 0.1, 0.3, and 0.5). For each figure, **(A)** plots the percent sample size was reduced and **(B)-(E)** plot performance measures (mean with 95% Monte Carlo confidence interval) for each method against the probability of item-level missingness ($p_{item}$ = 0.1, 0.3, 0.5, and 0.7), for each missingness mechanisms (MAR and MNAR). Specifically, **(B)** shows the percent bias of the mean FTND; **(C)** shows the percent bias of the standard error of the mean FTND compared to the empirical standard error for each method, as a measure of precision. For $n_{obs}$ = 52, **(D)** shows the bias of the regression coefficient for the total FTND score in single regression on the explanatory variable that recorded the answer to the question, ‚ÄúIs smoking allowed in your home?‚Äù with responses 0 = No and 1 = Yes; for $n_{obs}$ = 788, **(D)** shows the percent bias of that regression coefficient. The reason for this difference is that we prefer to show the percent bias when possible, because it is more interpretable; however, for the small sample size, the regression coefficient cannot be differentiated from zero, so calculating the percent bias results in dividing by numbers very close to zero, which makes it not interpretable. **(E)** shows the percent bias of the standard error of that regression coefficient compared to the empirical standard error for each method, as a measure of precision.

\

\

**Abbreviations:**\

CCA: complete case analysis\

FTND: Fagerstr√∂m Test for Nicotine Dependence\

HR: half-rule\

MAR: missing at random\

MNAR: missing not at random\

$n_{obs}$: sample size\

$p_{item}$: probability of item-level missingness\

$p_{sub}$: probability of subject-level missingness\

regr. coeff.: regression coefficient\

SE: standard error

```{r fig.width = 8.25,fig.height = 10.75}

library(tidyverse)

library(cowplot) # for nice plots in grid

fnames <- Sys.glob("sim_results/*.rds")

simres.df <- NULL

for (fname in fnames){

# Read data file

m.df <- read_rds(fname)

# Split filename into parts for parsing

spl <- str_split(fname, "_")

# If any NA (incl. NaN and -Inf, Inf), print where

if (sum(is.na(m.df)) > 0){

print("WARNING: NAs in")

print(fname)

print(which(is.na(m.df)))}

newrow <- m.df %>%

# Calculate summary statistics

summarise(across(everything(), list(mean=~mean(.,na.rm=TRUE), sd=~sd(.,na.rm=TRUE),

lb=~quantile(.,probs=c(0.025),na.rm=TRUE),

ub=~quantile(.,probs=c(0.975),na.rm=TRUE), median=~median(.,na.rm=TRUE)))) %>%

# Record simulation parameters (be careful about this if I rename files in FTND_sim_ASHLine.R)

mutate(nrep = as.integer(spl[[1]][3]), nobs = as.integer(spl[[1]][5]), psub=as.numeric(spl[[1]][7]), pitem=as.numeric(spl[[1]][9]))

# Append this simulation result

simres.df <- simres.df %>% rbind(newrow)

}

simres_long.df <- simres.df %>%

pivot_longer(

cols = -c(nrep, nobs, psub, pitem),

names_to = c("miss_mech", "method", "pop_or_indiv", "bias_or_prec", ".value"),

names_pattern = "FTND_?(.*)_(.*)_(.*)_(.*)_(.*)"

)

# Add labels to missingness mechanism etc. so that they appear nicer in plots

simres_long.df$miss_mech <- factor(simres_long.df$miss_mech,

levels = c("mcar", "mar", "mnar"),

labels = c("MCAR", "MAR", "MNAR"))

simres_long.df$method <- factor(simres_long.df$method,

levels = c("cca", "dropone", "itemmean", "itemmeanhr", "proration", "hotdeck"),

labels = c("CCA", "Drop One", "Item Mean", "HR Item Mean", "Proration", "Hot Deck"))

simres_long.df$pop_or_indiv <- factor(simres_long.df$pop_or_indiv,

levels = c("pop", "indiv"),

labels = c("Population", "Individual"))

simres_long.df$bias_or_prec <- factor(simres_long.df$bias_or_prec,

levels = c("meanbias", "meanbiasperc", "mean","meanse", "deltaN", "deltaNperc",

"regrcoeffbias", "regrcoeffbiasperc", "regrcoeffmean", "regrcoeffse",

"sebias", "sebiasperc", "regrcoeffsebias", "regrcoeffsebiasperc",

"meansegold", "regrcoeffmeansegold"),

labels = c("Bias of mean FTND", "% bias of mean FTND", "mean of FTND", "SE of mean FTND", "n_obs - n_method", "% sample size reduced",

"Bias of regression coeff.", "% bias of regression coeff.", "Regression coeff.", "SE of regression coeff.",

"Bias of SE of mean FTND", "% bias of SE of mean FTND", "Bias of SE of regression coeff.", "% bias of SE of regression coeff.",

"Empirical SE of mean FTND", "Empirical SE of regression coeff."))

# theme for plots in paper

theme_paper <- function(){

theme_bw() %+replace% #replace elements we want to change

theme(text = element_text(size = 14),

axis.title.y = element_text(angle = 90, vjust = 2, size = rel(1.2)),

axis.title.x = element_text(vjust = -0.2, size = rel(1.5)),

axis.text = element_text(size = rel(1.3)),

axis.line = element_line(colour = "black"),

axis.ticks = element_line(colour = "black"),

plot.margin = unit(c(t=0, r=0.1, b=-0.8, l=0.7), "cm"),

legend.text = element_text(size = rel(1.3)),

legend.title = element_text(size = rel(1.5)),

)

}

# Plots of bias of mean etc. vs. pitem,

# facet_wrap over miss mech, for various nobs & psub

# XXX: For debugging

# n_obs_vector <- c(788)

# frac_miss_sub_vector <- c(0.50)

n_obs_vector <- c(52, 788)

frac_miss_sub_vector <- c(0.10, 0.30, 0.50)

for (i in seq_along(n_obs_vector)){

for (j in seq_along(frac_miss_sub_vector)){

n_obs <- n_obs_vector[i]

p_sub <- frac_miss_sub_vector[j]

# position dodge to put different methods next to each other

pd <- position_dodge(width = 0.07)

data.df <- simres_long.df %>%

filter(psub == p_sub, nobs == n_obs) %>%

filter(miss_mech != "MCAR") # do not plot MCAR data

pA <- data.df %>%

filter(bias_or_prec == "% sample size reduced") %>%

ggplot(aes(x=pitem, y=mean, group = method, color = method)) +

geom_line(position = pd) +

geom_errorbar(aes(ymin = lb, ymax = ub, group = method, color = method), position = pd, width = 0.05, alpha = 0.8) +

geom_hline(yintercept = 0) +

geom_point(position = pd) +

scale_x_continuous(breaks=c(0.1, 0.3, 0.5, 0.7)) +

labs(y = "% sample size reduced",

color = "Method",

) +

facet_wrap("miss_mech", ncol = 1) +

theme_paper() +

theme(

axis.text.x=element_blank(),

axis.title.x=element_blank()#,

#plot.margin = unit(c(t=0, r=0, b=-0.8, l=0.5), "cm")

)

pB <- data.df %>%

filter(bias_or_prec == "% bias of mean FTND") %>%

ggplot(aes(x=pitem, y=mean, group = method, color = method)) +

geom_line(position = pd) +

geom_errorbar(aes(ymin = lb, ymax = ub, group = method, color = method), position = pd, width = 0.05, alpha = 0.8) +

geom_hline(yintercept = 0) +

geom_point(position = pd) +

scale_x_continuous(breaks=c(0.1, 0.3, 0.5, 0.7)) +

labs(y = "% bias of mean FTND",

color="Method",

) +

facet_wrap("miss_mech", ncol = 1, scales = "free_y") +

theme_paper() +

theme(

axis.text.x=element_blank(),

axis.title.x=element_blank()

)

pC <- data.df %>%

filter(bias_or_prec == "% bias of SE of mean FTND") %>%

ggplot(aes(x=pitem, y=mean, group = method, color = method)) +

geom_line(position = pd) +

geom_errorbar(aes(ymin = lb, ymax = ub, group = method, color = method), position = pd, width = 0.05, alpha = 0.8) +

geom_hline(yintercept = 0) +

geom_point(position = pd) +

scale_x_continuous(breaks=c(0.1, 0.3, 0.5, 0.7)) +

labs(y = "% bias of SE of mean FTND",

color="Method",

) +

facet_wrap("miss_mech", ncol = 1) +

theme_paper() +

theme(

axis.text.x=element_blank(),

axis.title.x=element_blank()

)

pDa <- data.df %>%

filter(bias_or_prec == "Bias of regression coeff.") %>%

ggplot(aes(x=pitem, y=mean, group = method, color = method)) +

geom_line(position = pd) +

geom_errorbar(aes(ymin = lb, ymax = ub, group = method, color = method), position = pd, width = 0.05, alpha = 0.8) +

geom_hline(yintercept = 0) +

geom_point(position = pd) +

scale_x_continuous(breaks=c(0.1, 0.3, 0.5, 0.7)) +

labs(x = bquote(p[item]),

y = "Bias of regr. coeff.",

color="Method",

) +

facet_wrap("miss_mech", ncol = 1) +

theme_paper()

pDb <- data.df %>%

filter(bias_or_prec == "% bias of regression coeff.") %>%

ggplot(aes(x=pitem, y=mean, group = method, color = method)) +

geom_line(position = pd) +

geom_errorbar(aes(ymin = lb, ymax = ub, group = method, color = method), position = pd, width = 0.05, alpha = 0.8) +

geom_hline(yintercept = 0) +

geom_point(position = pd) +

scale_x_continuous(breaks=c(0.1, 0.3, 0.5, 0.7)) +

labs(x = bquote(p[item]),

y = "% bias of regr. coeff.",

color="Method",

) +

facet_wrap("miss_mech", ncol = 1) +

theme_paper()

# if n_obs = 52, plot bias of regr. coeff.

# if n_obs = 788, plot % bias of regr. coeff.

pD <- pDa

if (n_obs == 788) {pD <- pDb}

pE <- data.df %>%

filter(bias_or_prec == "% bias of SE of regression coeff.") %>%

ggplot(aes(x=pitem, y=mean, group = method, color = method)) +

geom_line(position = pd) +

geom_errorbar(aes(ymin = lb, ymax = ub, group = method, color = method), position = pd, width = 0.05, alpha = 0.8) +

geom_hline(yintercept = 0) +

geom_point(position = pd) +

scale_x_continuous(breaks=c(0.1, 0.3, 0.5, 0.7)) +

labs(x = bquote(p[item]),

y = "% bias of SE of regr. coeff.",

color="Method",

) +

facet_wrap("miss_mech", ncol = 1) +

theme_paper()

plot_title <- ggdraw() +

draw_label(bquote(list(n[obs] == .(n_obs), p[sub] == .(p_sub))),

fontface = 'bold', x = 0, hjust = 0, size = 24) +

theme(

# add margin on the left of the drawing canvas,

# so title is aligned with left edge of first plot

plot.margin = margin(0, 0, 0, 80)

)

# extract the legend from one of the plots

legend <- get_legend(

# create some space around the legend if needed

pA + theme(legend.box.margin = margin(0, 0, 0, 0))

)

plot_plots <- plot_grid(pA + theme(legend.position="none"), NULL,

pB + theme(legend.position="none"),

pC + theme(legend.position="none"),

pD + theme(legend.position="none"),

pE + theme(legend.position="none"),

labels = c('(A)', '', '(B)', '(C)', '(D)', '(E)'),

label_size = 14,

hjust = -0.4,

vjust = 1.0,

nrow = 3,

ncol = 2,

align = "hv")

p <- plot_grid(plot_title, plot_plots, "", ncol = 1,

# rel_heights values control vertical title margins

rel_heights = c(0.05, 1, 0.04)

) + draw_grob(legend, x=0.3, y = 0.33)

figname <- paste0("figures/nobs_", n_obs_vector[i], "_psub_", frac_miss_sub_vector[j], ".png")

# need to comment out the png commands of png() and dev.off() if want to output figs to pdf

#png(figname, width = 8.25, height = 10.75, units = "in", res = 600)

plot(p) # need to explicitly print the ggplot when in a for loop

#dev.off()

}

}

# Use code like the following to get out max and min results to put in text of paper:

# simres_long.df %>% filter(bias_or_prec == "% bias of SE of regression coeff.", nobs ==788, method == "CCA") %>% arrange(mean) %>% View()

# simres_long.df %>% filter(bias_or_prec == "% bias of SE of regression coeff.", nobs ==788, method == "Drop One") %>% arrange(mean) %>% View()

# simres_long.df %>% filter(bias_or_prec == "% bias of SE of regression coeff.", nobs ==788, method == "Item Mean") %>% arrange(mean) %>% View()

# simres_long.df %>% filter(bias_or_prec == "% bias of SE of regression coeff.", nobs ==788, method == "HR Item Mean") %>% arrange(mean) %>% View()

# simres_long.df %>% filter(bias_or_prec == "% bias of SE of regression coeff.", nobs ==788, method == "Proration") %>% arrange(mean) %>% View()

# simres_long.df %>% filter(bias_or_prec == "% bias of SE of regression coeff.", nobs ==788, method == "Hot Deck") %>% arrange(mean) %>% View()

```
