# Supplementary material for: Managing missing items in the Fagerström Test for Nicotine Dependence: a simulation study
Source: BMC Med Res Methodol. 2022 May 20;22:145. doi: 10.1186/s12874-022-01637-2 (PMC9121580; doi:10.1186/s12874-022-01637-2)
Supplement: Supplementary file 6 — Additional file 6. Plots of FTND simulation results. [file 12874_2022_1637_MOESM6_ESM.pdf]

Additional File 6: Plots of FTND simulation results

"Managing missing items in the Fagerström Test for Nicotine Dependence: a simulation study"

Shannon L Gutenkunst & Melanie L Bell

There is a separate figure for each combination of sample size (n_{obs} = 52 and 788) and probability of subject-level missingness (p_{sub} = 0.1, 0.3, and 0.5). For each figure, **(A)** plots the percent sample size was reduced and **(B)-(E)** plot performance measures (mean with 95% Monte Carlo confidence interval) for each method against the probability of item-level missingness (p_{item} = 0.1, 0.3, 0.5, and 0.7), for each missingness mechanisms (MAR and MNAR). Specifically, **(B)** shows the percent bias of the mean FTND; **(C)** shows the percent bias of the standard error of the mean FTND compared to the empirical standard error for each method, as a measure of precision. For n_{obs} = 52, **(D)** shows the bias of the regression coefficient for the total FTND score in single regression on the explanatory variable that recorded the answer to the question, "Is smoking allowed in your home?" with responses 0 = No and 1 = Yes; for n_{obs} = 788, **(D)** shows the percent bias of that regression coefficient. The reason for this difference is that we prefer to show the percent bias when possible, because it is more interpretable; however, for the small sample size, the regression coefficient cannot be differentiated from zero, so calculating the percent bias results in dividing by numbers very close to zero, which makes it not interpretable. **(E)** shows the percent bias of the standard error of that regression coefficient compared to the empirical standard error for each method, as a measure of precision.

Abbreviations:
CCA: complete case analysis
FTND: Fagerström Test for Nicotine Dependence
HR: half-rule
MAR: missing at random
MNAR: missing not at random
n_{obs}: sample size
p_{item}: probability of item-level missingness
p_{sub}: probability of subject-level missingness
regr. coeff.: regression coefficient
SE: standard error

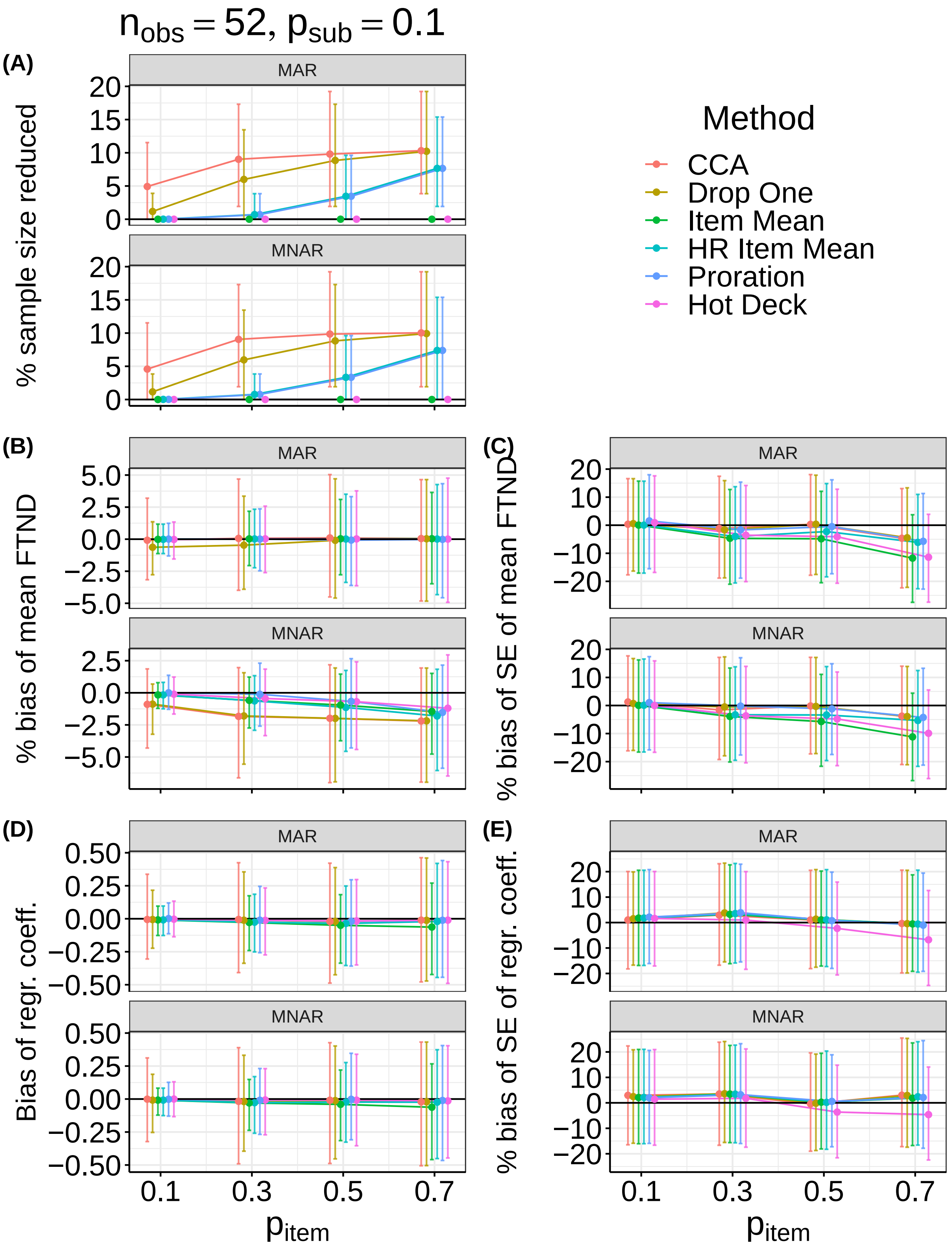

$n_{obs} = 52, p_{sub} = 0.1$
(A)
MAR
MNAR
% sample size reduced
Method
CCA
Drop One
Item Mean
HR Item Mean
Proration
Hot Deck
(B)
% bias of mean FTND
(C)
% bias of SE of mean FTND
(D)
Bias of regr. coeff.
(E)
% bias of SE of regr. coeff.
0.1
0.3
0.5
0.7
p_{item}

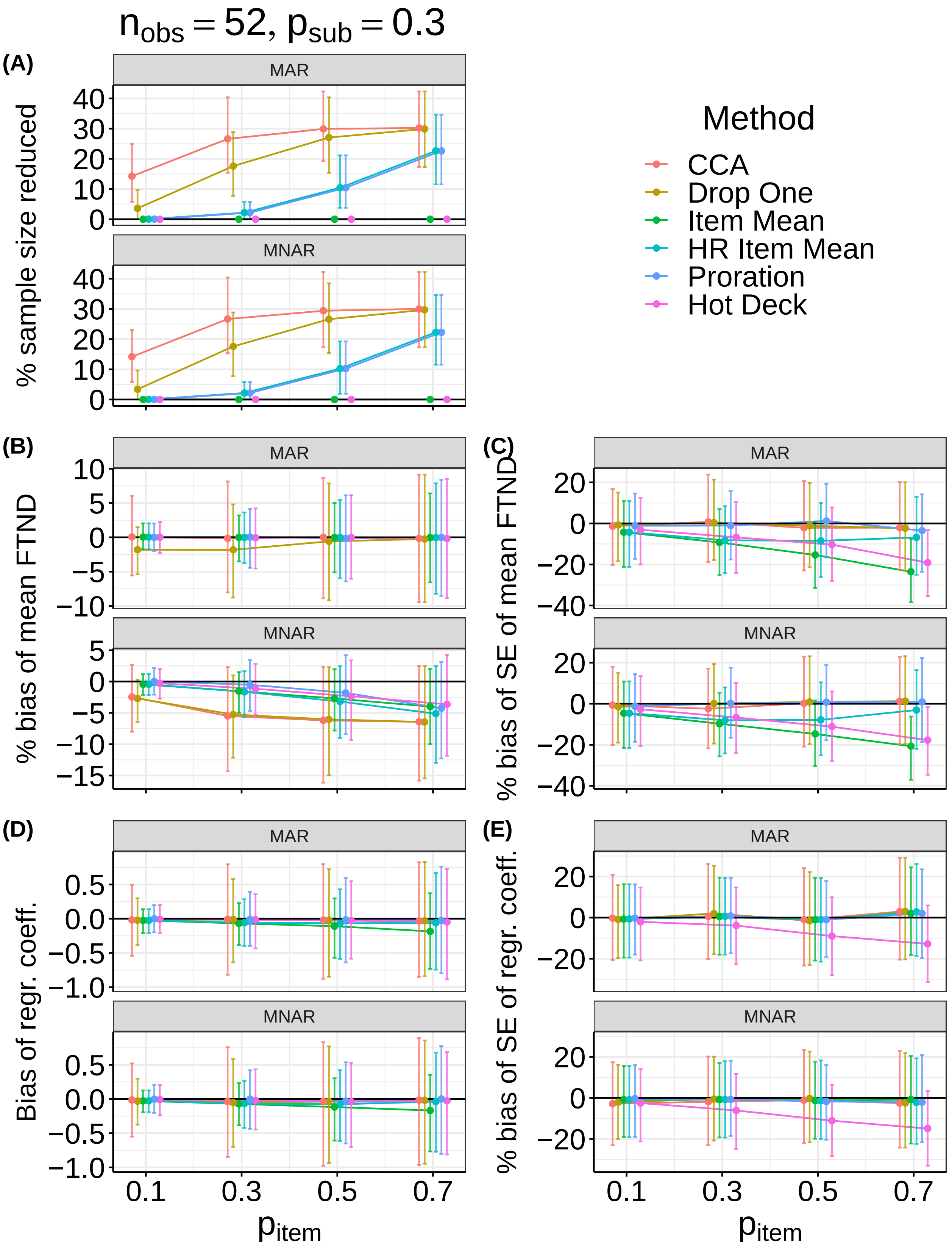

$n_{obs} = 52, p_{sub} = 0.3$
(A)
(B)
(C)
(D)
(E)
MAR
MNAR
% sample size reduced
% bias of mean FTND
% bias of SE of mean FTND
Bias of regr. coeff.
% bias of SE of regr. coeff.
p_{item}
Method
CCA
Drop One
Item Mean
HR Item Mean
Proration
Hot Deck

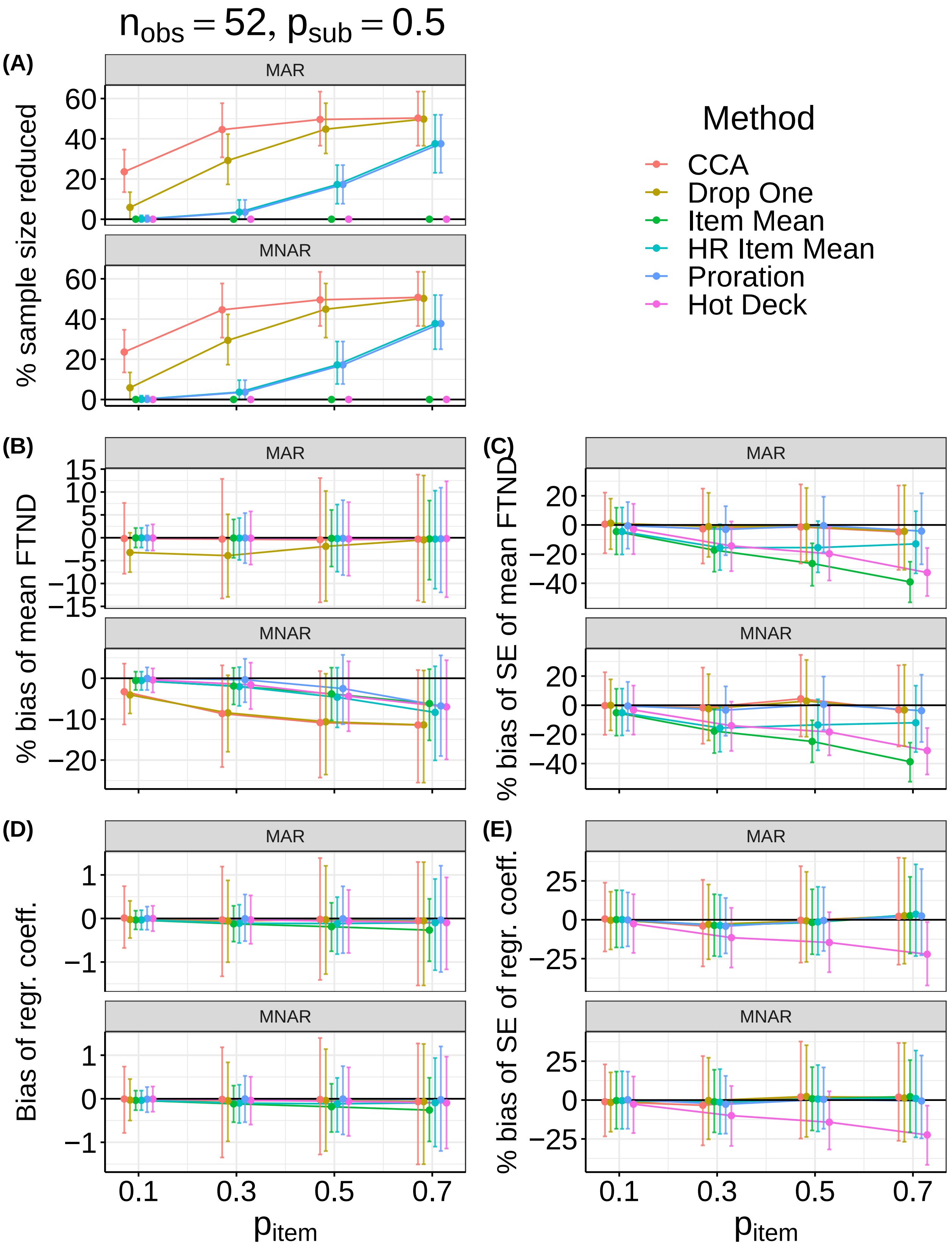

$n_{obs} = 52, p_{sub} = 0.5$
(A)
MAR
MNAR
% sample size reduced
60
40
20
0
Method
CCA
Drop One
Item Mean
HR Item Mean
Proration
Hot Deck
(B)
% bias of mean FTND
15
10
5
0
−5
−10
−15
−20
(C)
% bias of SE of mean FTND
20
0
−20
−40
(D)
Bias of regr. coeff.
1
0
−1
(E)
% bias of SE of regr. coeff.
25
0
−25
0.1
0.3
0.5
0.7
p_{item}

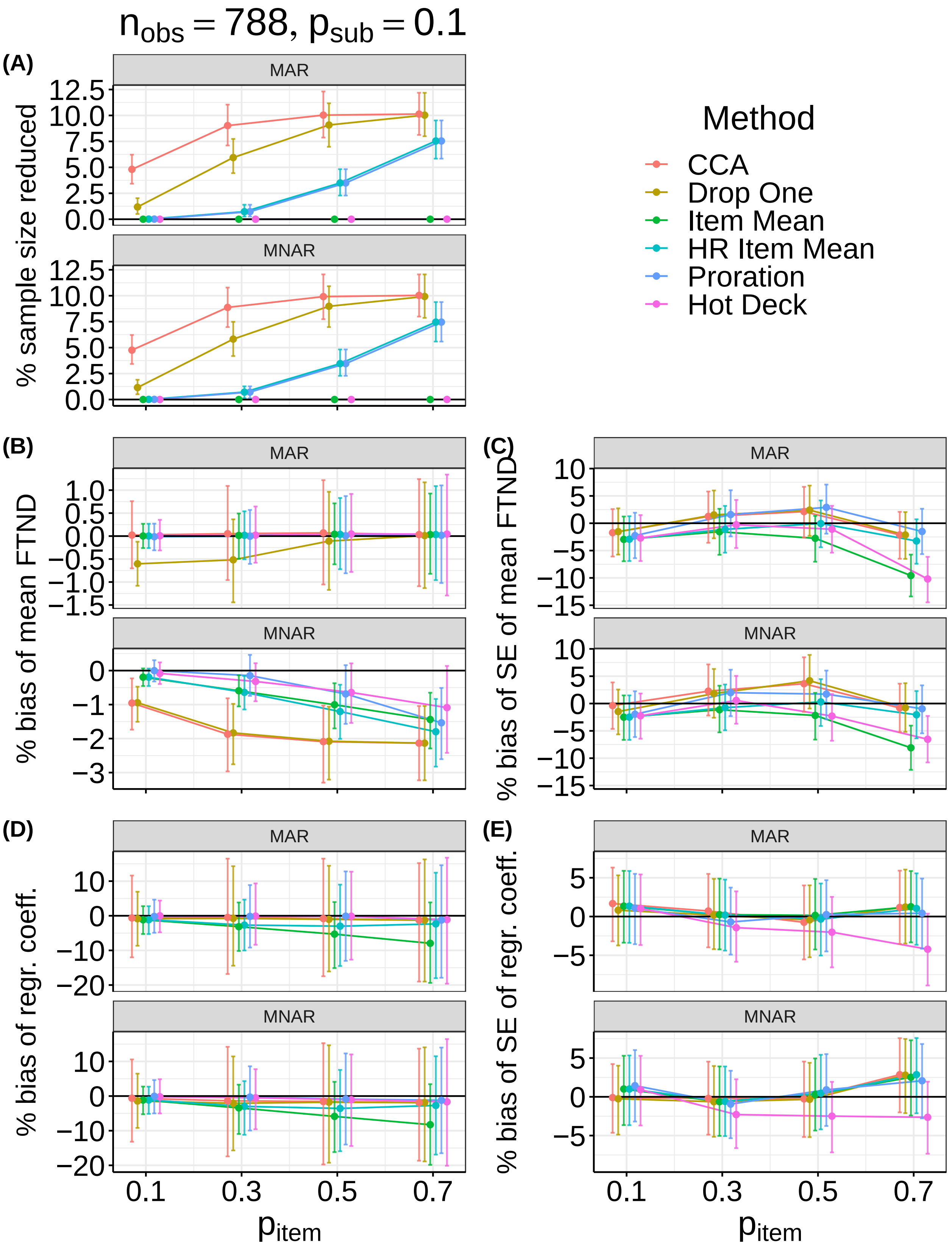

$n_{obs} = 788$, $p_{sub} = 0.1$
(A)
MAR
MNAR
% sample size reduced
(B)
% bias of mean FTND
(C)
% bias of SE of mean FTND
(D)
% bias of regr. coeff.
(E)
% bias of SE of regr. coeff.
p_{item}
Method
CCA
Drop One
Item Mean
HR Item Mean
Proration
Hot Deck

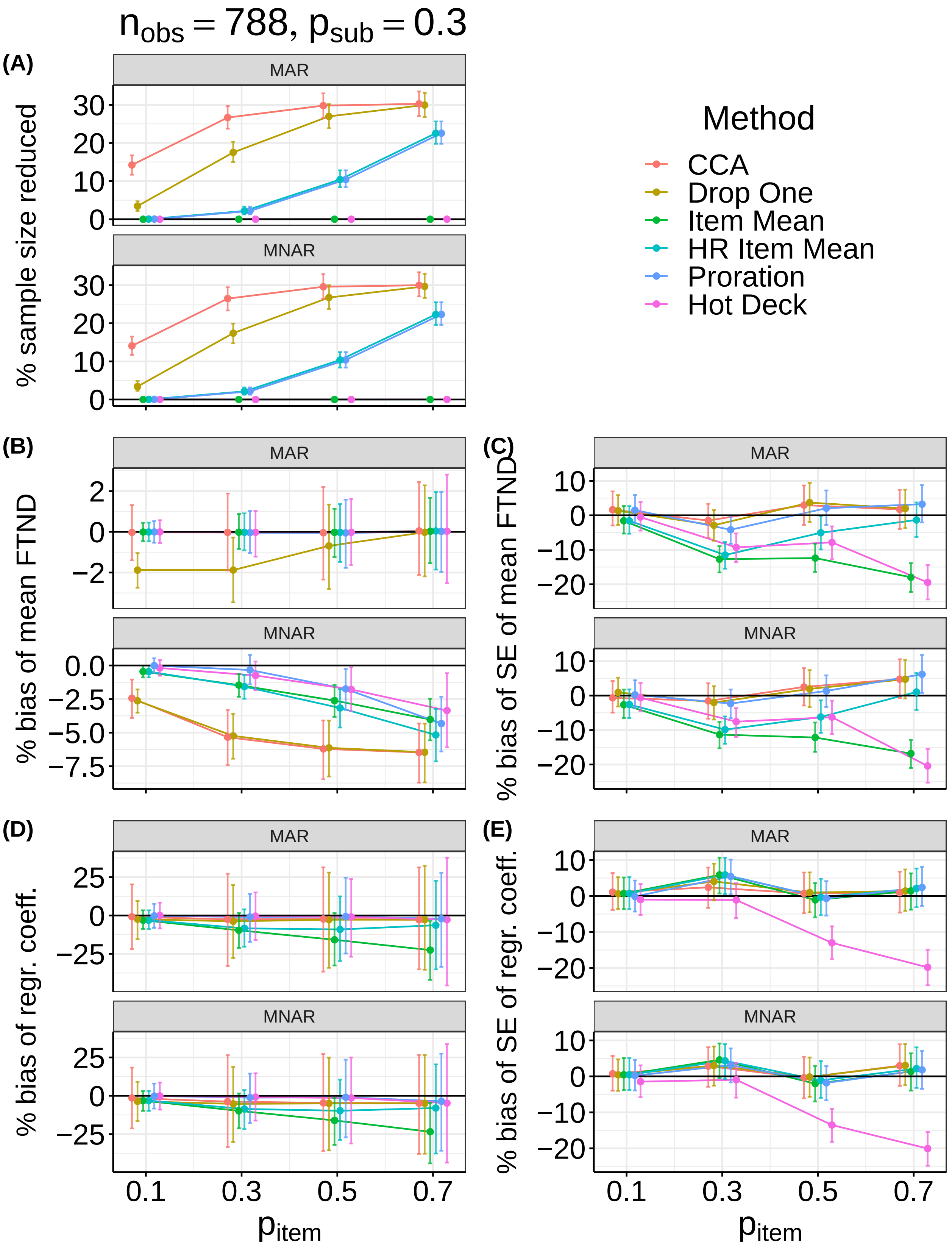

$n_{obs} = 788, p_{sub} = 0.3$
(A)
MAR
MNAR
% sample size reduced
(B)
% bias of mean FTND
(C)
% bias of SE of mean FTND
(D)
% bias of regr. coeff.
(E)
% bias of SE of regr. coeff.
Method
CCA
Drop One
Item Mean
HR Item Mean
Proration
Hot Deck
0.1
0.3
0.5
0.7
p_{item}

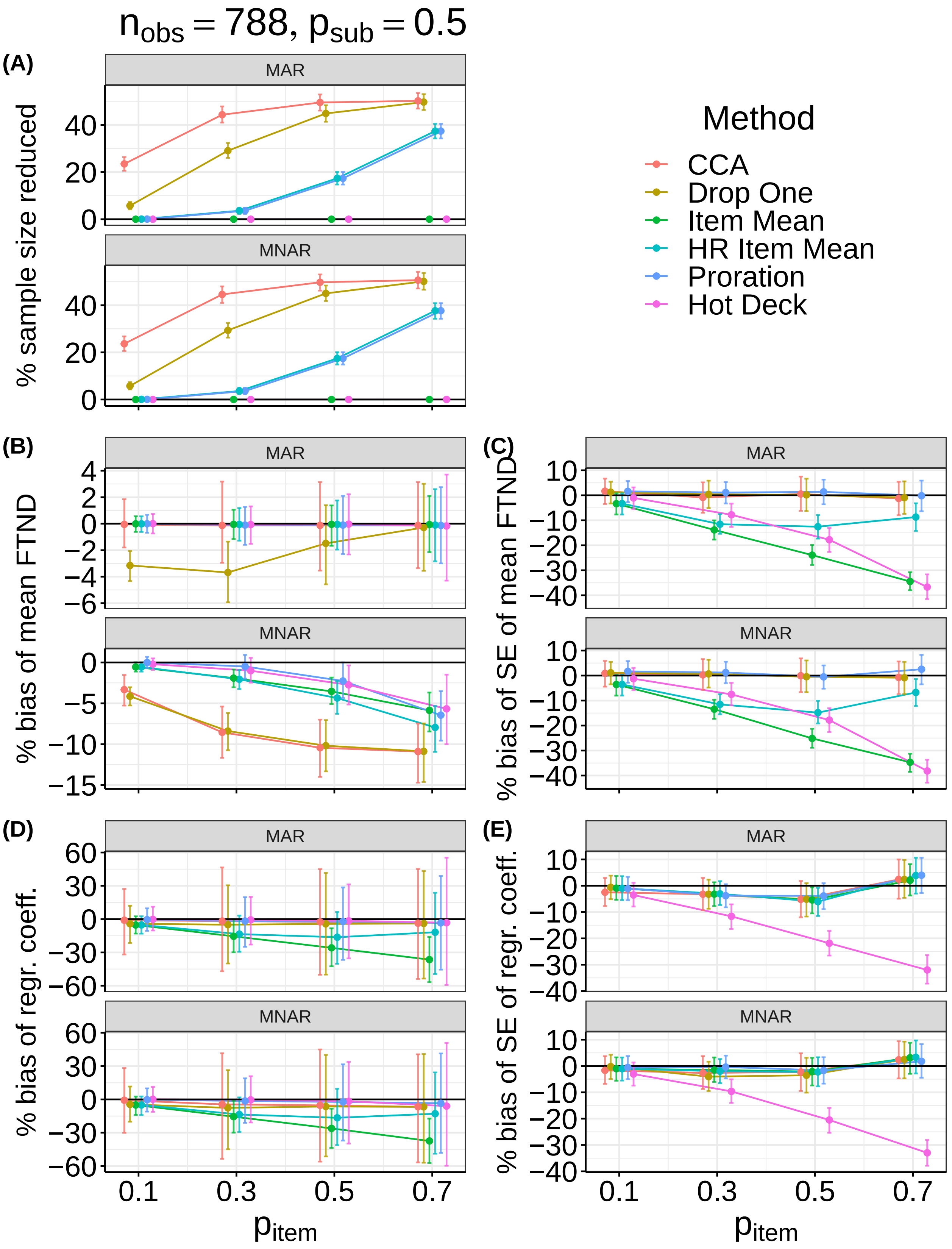
$n_{obs} = 788$, $p_{sub} = 0.5$
(A)
(B)
(C)
(D)
(E)
MAR
MNAR
% sample size reduced
% bias of mean FTND
% bias of SE of mean FTND
% bias of regr. coeff.
% bias of SE of regr. coeff.
p_{item}
Method
CCA
Drop One
Item Mean
HR Item Mean
Proration
Hot Deck
